# Coding Capacity of Purkinje Cells With Different Schemes of Morphological Reduction

**DOI:** 10.3389/fncom.2019.00029

**Published:** 2019-05-15

**Authors:** Lingling An, Yuanhong Tang, Quan Wang, Qingqi Pei, Ran Wei, Huiyuan Duan, Jian K. Liu

**Affiliations:** ^1^School of Computer Science and Technology, Xidian University, Xi'an, China; ^2^Department of Neuroscience, Psychology and Behaviour, Centre for Systems Neuroscience, University of Leicester, Leicester, United Kingdom

**Keywords:** Purkinje cell, neuronal morphology, dendritic model, rate coding, temporal coding, multi-compartmental models

## Abstract

The brain as a neuronal system has very complex structures with a large diversity of neuronal types. The most basic complexity is seen from the structure of neuronal morphology, which usually has a complex tree-like structure with dendritic spines distributed in branches. To simulate a large-scale network with spiking neurons, the simple point neuron, such as the integrate-and-fire neuron, is often used. However, recent experimental evidence suggests that the computational ability of a single neuron is largely enhanced by its morphological structure, in particular, by various types of dendritic dynamics. As the morphology reduction of detailed biophysical models is a classic question in systems neuroscience, much effort has been taken to simulate a neuron with a few compartments to include the interaction between the soma and dendritic spines. Yet, novel reduction methods are still needed to deal with the complex dendritic tree. Here, using 10 individual Purkinje cells of the cerebellum from three species of guinea-pig, mouse and rat, we consider four types of reduction methods and study their effects on the coding capacity of Purkinje cells in terms of firing rate, timing coding, spiking pattern, and modulated firing under different stimulation protocols. We found that there is a variation of reduction performance depending on individual cells and species, however, all reduction methods can preserve, to some degree, firing activity of the full model of Purkinje cell. Therefore, when stimulating large-scale network of neurons, one has to choose a proper type of reduced neuronal model depending on the questions addressed. Among these reduction schemes, Branch method, that preserves the geometrical volume of neurons, can achieve the best balance among different performance measures of accuracy, simplification, and computational efficiency, and reproduce various phenomena shown in the full morphology model of Purkinje cells. Altogether, these results suggest that the Branch reduction scheme seems to provide a general guideline for reducing complex morphology into a few compartments without the loss of basic characteristics of the firing properties of neurons.

## 1. Introduction

A single neuron is thought to be the basic computation unit in the complex neuronal system. Understanding the computations and dynamics of single neurons is an integral part of explaining their functional roles within a large neural network. However, there is a complex dendritic structure for a single neuron's morphology. The question is, what are the key properties of neuronal morphology for understanding a complex process such as information processing and dynamic behavior that emerges from a network of neurons? How can one reduce the whole morphology, but still keep these essential dynamics? To address these questions, a number of methods and models have been developed to describe firing properties of single neurons at different levels of abstractions and reductions. A simple, perhaps the most compact version of a neuronal model, is the so-called integrate-and-fire (IAF) model (Lapicque, 1907). The IAF model of neuronal dynamics is considered to be a simple way to explain certain aspects of neuronal behaviors (Burkitt, 2006a,b). In particular, it has been used mostly for the simulation of large-scale neuronal networks. However, with the advancement of experimental techniques in characterizing fine dynamics of neurons, there is an increasing volume of evidence showing that the IAF approach cannot capture the dynamics and computations of real neurons (Mainen and Sejnowski, 1996; Koch, 1999; Poirazi et al., 2003; Ostojic et al., 2015; Amsalem et al., 2018), in which neuronal morphology, ion channels, and synapse distributions, all affect the activity of neurons (Herz et al., 2006). The classical Rall cable theory is useful to help understand the contribution of the neuronal spatial structure in its function and dynamics (Rall, 1959). By extending the Rall cable theory, the real neuronal morphology can be reconstructed based on the anatomy to ensure that its electrophysiological characteristics are unchanged. Although the detailed model can describe the dynamics of individual neurons very well, their high dimensionality and complex spatial structure makes the calculation expensive and unsuitable for large-scale network simulations.

The classical Hodgkin-Huxley single-compartment model ignores the spatial structure of neurons to mainly explain the ionic mechanisms and how action potentials are initiated and propagated in neurons (Hodgkin and Huxley, 1990). However, recent theoretical work shows that the single somatic point neuron is not enough to capture the detailed firing activities of Purkinje cells observed in experiments (Ostojic et al., 2015). The neuronal model with at least two compartments of soma and dendrite is necessary. Similarly, the reduced model with only one or a few dendritic compartments is usually sufficient to understand detailed neuronal activities (Brown et al., 2011; Armin et al., 2012; Marasco et al., 2013; Amsalem et al., 2018). For large-scale network studies, reduced models provide a good balance between biological activity and computational efficiency (Herz et al., 2006). However, what reduction schemes, that simplify the whole dendritic morphology, is still not well understood (Marasco et al., 2013; Amsalem et al., 2018)?

Here we address this question by focusing on the Purkinje cells (PCs) of the cerebellum. The cerebellum, as one of the most well-studied brain areas, traditionally plays an essential role in motor control (Ito, 1984; Zeeuw et al., 1997; Amir and Zee, 2011; Manto et al., 2012) and learns of vestibular-ocular reflex (Du et al., 1995; Blazquez et al., 2004; Hirata et al., 2012) and eyelid reflex regulation (Koekkoek et al., 2003; Jiménezdaz et al., 2004). In recent years, a large number of studies have shown that the cerebellum is also involved in the processing of information such as cognition, language, attention, and memory (Ito, 2008; Strick et al., 2009; Wolf et al., 2009; Tsai et al., 2012; Wagner et al., 2017; Bostan and Strick, 2018; Raymond and Medina, 2018).

PCs, as the only output neurons in the cerebellum, is an indispensable component in the mechanism of synaptic plasticity in cerebellar learning. PCs receive parallel fiber (PF) input that generates high-frequency simple spikes (SSs) to predict ongoing movements (Loewenstein et al., 2005). The high-frequency SS discharge of PCs encodes information about movement, including performance errors and kinematics (Robinson and Fuchs, 2001; Popa et al., 2016). Importantly, SS modulation both leads and lags behavior, which means that individual PCs may carry predictive and feedback signals about motor commands and corresponding behaviors (Hewitt et al., 2015; Popa et al., 2015; Chen et al., 2016; Streng et al., 2018). PC has a tree-like morphology structure with an intricately elaborate dendritic arbor, which can be characterized by a large number of dendritic spines distributed in its dendritic branches. Given such a sophisticated dendritic structure, it is necessary to find a suitable level of abstraction with a reduced morphological structure to understand the working mechanisms of PC for the integration of PF synaptic inputs.

In a previous study (Marasco et al., 2013), a reduction method based on the Strahler analysis of neuron morphology was applied to PCs with an arbitrary dendritic distribution of ion channels and synaptic inputs and without any fitting or tuning procedures. In particular, this reduction method can ensure that the somatic membrane potential trace is accurate while reducing the runtime of the simulation significantly. In this study, we systematically modeled the morphology of PCs from three species of guinea-pigs, mice and rats, and investigated the effect of four different reduction schemes on the coding capacity of PCs, in terms of firing rate, spike timing, spike pattern, and modulation of firing amplitude and phase, with several types of stimulation protocols of PF synaptic inputs, including the Poisson and modulated renewal process.

We found that the PC firing rate coding, which is the input-output relationship of the PC firing activity under different frequencies, is slightly different in low frequency stimuli but significantly different in high frequency stimuli at a millimeter-scale reaction under different reduction schemes of morphology. The PC spike timing coding quantified by the inter spike intervals and regular/irregular spike patterns are well captured by different reduction schemes. In addition, for a modulated stimulus with a frequency following a sinusoidal modulation, the phase and amplitude of the PC response curve is also well described by different reduction schemes. However, there is a variation of coding performance across different reduction schemes for PCs from different species. Among these reduction schemes, the Branch method, which keeps the feature of the geometrical volume of a neuron, can achieve a good balance between different performance measures of accuracy, simplification, computational efficiency, and spike shape change of morphology reduction, and reproduce various phenomena shown in the full model with the whole PC morphology. Thus, these results suggest that the Branch reduction scheme could serve as a general guideline to reduce complex morphology into a few compartments without the loss of basic characteristics of the firing property of neurons.

## 2. Methods

### 2.1. Neuronal Morphology Reduction

**Figure 1:**
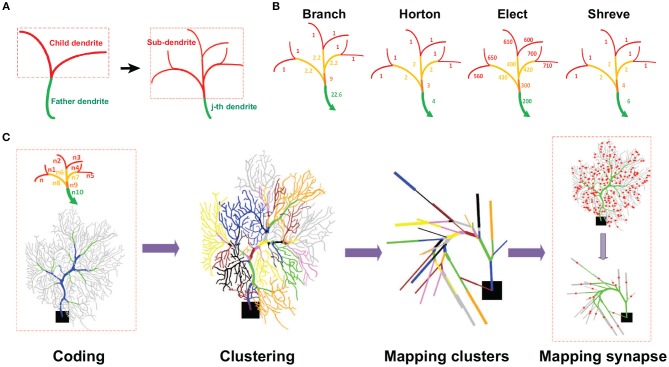
Schematic view of reduction process. **(A)** Illustration of a dendritic field. Each sub-branch has a father dendrite with several child dendrites. Each of them can be indexed as the *j*-*th* dendrite (green) with a set of sub-dendrites (red). **(B)** Illustration of different coding schemes of a neuronal morphological structure. Levels of dendrites are colored differently with terminal dendrites as red and non-terminal dendrites as other colors. The number of each dendrite is the order value obtained by different coding schemes. For instance, for the Branch model, the order value of non-terminal dendrites is the sum of all their sub-dendrite order values plus a weight as the number of sub-dendrites divided by 10. Thus, the order values of all terminal dendrites are 1. At the next level, the dendrites have an order value of 2.2 that is the sum of 2, from all their sub-dendrite order values, with 0.2 as a weight of the number of sub-dendrites divided by 10. So, the final order value at this level is 2.2. This process moves from the terminal or non-terminal dendrites level by level to obtain all the order values. Note Elect method operates in an opposite way. **(C)** Reduction process. Coding: The first step is to encode the morphological structure and distinguish different functional areas (Blue, Trunk. Green, Smooth. Gray, Spiny) of a neuron. Clustering: the second step is to set clusters according to the coding number. Mapping clusters: the third step is to map each cluster into a single compartment. Mapping synapses: the fourth step is to map synaptic locations in the reduced model. The red dots indicate synaptic locations in the spiny dendrites.

A reduction method maps the full morphological structure into an equivalent reduced model with much fewer dendritic compartments. As a typical tree-like structure, one has to identify the father and child dendrites, and mark them with a set of graphic notations (see Figure 1A). For the markers, one needs to set up a coding scheme such that there is an order value for each selected section or area as illustrated in Figure 1B. Here we proposed four different coding schemes where the order values are determined differently. The motivations of these coding schemes are inspired by a general analysis of river networks (Horton, 1945; Shreve, 1967).

The main feature of the Shreve and Horton methods is to identify and classify river types based on the importance of rivers in water networks. Specifically, Shreve coding is to define a river without tributaries as level 1, and other river levels are obtained by adding their tributary levels. The characteristics of the Shreve method are similar to the calculation of confluence and have a relatively large reference value in some simulations of hydrological flow and sediment volume. Horton coding defines a river without tributaries as level 1, and other river levels are obtained by the maximum level of its tributary. Horton code mainly reflects the hierarchical relationship in the water network and the depth of the river subtree. The Purkinje cell dendritic structure is similar to the river network, and these two methods can be used to classify the dendrites and then merge and simplify the unimportant dendrites. As a result, the different functional regions of PCs can be characterized by their order values.

Before setting the order value, the PC tree-like morphological structure can be divided into terminal and non-terminal dendrites. We used *E* and *O* to represent the set of terminal dendrites and non-terminal dendrites, respectively. Specifically, *E* = {*e*_1_, *e*_2_, ··· , *e*_*i*_} is the terminal dendrites set, *e*_*i*_ is the *i*-*th* dendrite in the set *E*, *N*_*e,i*_ is the order value of the *i*-*th* dendrite in set *E*. *O* = {*o*_1_, *o*_2_, ..*o*_*j*_} is the non-terminal dendrites set, *o*_*j*_ is the *j*-*th* dendrite in *O*, *N*_*o,j*_ is the order value of the *j*-*th* dendrite in *O*. Then according to the order value *N*, *N* = *N*_*e,i*_ or *N* = *N*_*o,j*_, the dendrites can be classified as spiny, smooth, or trunk, as follows:

(1) $$\begin{array}{l} dend\in \{\begin{array}{cc} Spiny, & \text{if}\;N\leq s1\\ Smooth, & \text{if}\;s1<N<s2\\ Trunk, & \text{if}\;N\geq s2 \end{array} \end{array}$$

where *s*1 and *s*2 are the best threshold values for dividing the functional areas of spiny, smooth, and trunk dendrites. We tested several different values of *s*1 and *s*2, and determined the best values that can achieve the balance between a higher reduction and a better overall accuracy. These values, chosen for each PC are given in Table 1.

**Table 1:** Best thresholds of reduction models for each cell.

		**Guinea-pig1**	**Guinea-pig2**	**Guinea-pig3**	**Mouse1**	**Mouse2**	**Mouse3**	**Mouse4**	**Rat1**	**Rat2**	**Rat3**
Branch	s1	3	3	3	3	3	3	3	3	3	3
	s2	8	8	8	8	8	8	8	8	8	8
Horton	s1	3	3	3	3	3	3	3	3	3	3
	s2	30	90	20	50	50	30	30	20	30	20
Elect	s1	13	16	18	62	87	35	69	33	27	86
	s2	17	17	19	63	88	36	70	34	29	87
Shreve	s1	10	10	10	10	10	10	10	10	10	10
	s2	30	30	30	30	30	30	30	30	30	30

In this study, we used four different coding schemes as defined below.

#### 2.1.1. Branch Scheme

In this scheme, the order values of dendrites are determined by the branches of each dendrite. The order values of terminal dendrites are fixed as 1, i.e., *N*_*e,i*_ = 1, then the non-terminal dendrite order values are given as:

(2) $$\begin{array}{l} N_{o,j}=\overset{n}{\underset{k=1}{\sum }}N_{o,j_{k}}+weight \end{array}$$

*N* = {*N*_*o*,*j*_1__, *N*_*o*,*j*_2__, ···*N*_*o*,*j*_*n*__} is the order value of sub-dendrites of the *j*-*th* dendrite in set *O*, *j*_*k*_ is *k*-*th* sub-dendrite of the *j*-*th* dendrite, *n* is the number of the sub-dendrites of the *j*-*th* dendrite, and *weight* = *n*/10, where factor 10 is the best value for a trade-off between simplification and accuracy of the modeling in our case.

#### 2.1.2. Horton Scheme

In this scheme, the dendritic tree is encoded by Horton analysis (Horton, 1945). We set the order value for each section. Terminal dendrites of set *E* have the order value as 1, *N*_*e,i*_ = 1. The order value for non-terminal dendrites can be set as:

(3) $$\begin{array}{l} N_{o,j}=max\left(N_{o,j_{1}},N_{o,j_{2}},\cdot \cdot \cdot \;,N_{o,j_{n}}\right)+1 \end{array}$$

where, *N* = {*N*_*o*,*j*_1__, *N*_*o*,*j*_2__, ···*N*_*o*,*j*_*n*__} is the order value of sub-dendrites of the *j*-*th* dendrite in set *O*, *j*_*n*_ is *n*-*th* sub-dendrite of the *j*-*th* dendrite, and *n* is the number of the sub-dendrites of the *j*-*th* dendrite.

#### 2.1.3. Shreve Scheme

In this coding scheme, the morphological structure is quantitatively analyzed using the adaptive Shreve encoding scheme (Shreve, 1967). Terminal dendrites of set *E* are given by the order value as 1, *N*_*e,i*_ = 1. The non-terminal dendrites order values are given as:

(4) $$\begin{array}{l} N_{o,j}=\overset{n}{\underset{k=1}{\sum }}N_{o,j_{k}} \end{array}$$

where *N*_*o*,*j*_*k*__ is the order values of the *k*-*th* child dendrite of the *j*-*th* in set *O*, and *n* is the number of the child dendrites of the *j*-*th* dendrite.

#### 2.1.4. Elect Scheme

In this scheme, different dendritic geometries result in different electricity properties of dendrites, so one can use the feature of input resistance to analyze tree dendrites, so that the values of the input resistance of dendrites are used as the dendritic order values:

(5) $$\begin{array}{l} N=\left(N_{e,i},N_{o,j}\right)=\text{Input resistance} \end{array}$$

In this model, different functional regions of PCs can be characterized by their order values as

(6) $$\begin{array}{l} dend\in \{\begin{array}{cc} Spiny, & \text{if}\;N\geq s2\\ Smooth, & \text{if}\;s1<N<s2\\ Trunk, & \text{if}\;N\leq s1 \end{array} \end{array}$$

Note Equation (6) operates in the opposite way of Equation (1), since for Branch, Horton and Shreve methods, tree order values are increased from spiny to trunk, but the Elect method works as a decreasing process.

Once the coding stage is finished, the rest of the work-flow process of reducing neuronal morphology is illustrated in Figure 1C. One can collect those dendrites according to their order values into a cluster, such that there are three sets of clusters as *C*_*trunk*_ for trunk dendrites, *C*_*smooth*_ for smooth dendrites, and *C*_*spiny*_ for spiny dendrites. Within each set, there is a series of subsets of the cluster in each region of the dendritic field. Such a subset of clusters can be mapped into one single compartment by using the same merging rule as in Marasco et al. (2013), then ionic and synaptic conductances are scaled by a factor to preserve membrane area (Marasco et al., 2013) or preserve volume. To preserve the membrane area in Horton, Shreve, and Elect models, we used a factor $f_{s,j}^{eq}=\frac{\sum_{j}s_{j}}{s_{j}^{eq}},$ where ∑_*j*_*s*_*j*_ is the sum of the membrane area of each section in the clusters, and $s_{j}^{eq}$ is the membrane area of the equivalent compartment. However, in the Branch model, to preserve the volume instead of the membrane area, we used a factor $f_{v,j}^{eq}=\frac{\sum_{j}v_{j}}{v_{j}^{eq}}$, where ∑_*j*_*v*_*j*_ is the sum of the volumes of each section in the clusters, and $v_{j}^{eq}$ is the volume of the equivalent compartment. Finally, synapse locations are sequentially mapped in each reduced model in the same way as in Marasco et al. (2013).

### 2.2. Multi-Compartment Model of Purkinje Cell

To compare different reduced models with full morphology, we used multi-compartment models based on 10 detailed morphological 3D reconstructions of Purkinje cells of guinea-pigs, mice, and rats, from a public archive *neuromorpho.org*. Specific capacitance was set to 0.8*F*/*cm*^2^ in the soma, and 1.5*F*/*cm*^2^ in trunk dendrites, smooth dendrites, and spiny dendrites. Internal axial resistivity was set to 250 Ω/*cm* similar to the values used in Ostojic et al. (2015) and Rapp et al. (1994).

The same set of parameter values of passive properties, such as voltage-dependent ionic channels, kinetic, and distribution, was used for all morphologies of PCs. There are 13 different types of voltage-gated ion channels modeled, eight of which (P-type *Ca*^2+^ channel, T-type *Ca*^2+^ channel, class-E *Ca*^2+^ channel, persistent *K*^+^ channel, A-type *K*^+^ channel, D-type *K*^+^ channel, delayed rectifier, decay of sub-membrane *Ca*^2+^) were inserted into the soma and dendrites. In addition, three ion channels (fast and persistent sodium channel, anomalous rectifier channel) were solely added to the soma, and two ion channels (high-threshold calcium-activated potassium channel, low-threshold calcium-activated potassium channel) were solely added to the dendrites (Schutter and Bower, 1994a; Miyasho et al., 2001).

### 2.3. Stimulation Protocols

Stimulation of PC was implemented by parallel fiber (PF) inputs, where synaptic input from each PF to PC is characterized by AMPA receptors (Gao et al., 2012). Following the typical values estimated from experiments, a total of 1000 PF connections for a single PC was used as previously described (Masoli and DAngelo, 2017). AMPA synapses were inserted only in the spiny dendrites with a random distribution. AMPA synapses were modeled as a double exponential conductance change with 0.5 and 1.2 ms for rising and decay time, respectively (Schutter and Bower, 1994b), and the maximal synaptic conductance was drawn from a Gaussian distribution as 5 ± 0.5 nS.

In addition, for PCs affected by direct synaptic inhibition coming from molecular layer interneurons, we used a total of 500 inhibitory connections on a single PC. These synapses were randomly distributed on the spiny dendrites, and modeled as *GABA*_*A*_ (He et al., 2015) with a double exponential conductance change with 0.5 and 2.5 ms for rising and decay time, respectively, and the maximal synaptic conductance was drawn from a Gaussian distribution as 5±0.5 nS.

PCs aligned on the mediolateral axis receive about 175,000 PF inputs (Napper and Harvey, 1988; Hoxha et al., 2016). This arrangement results in the hypothesis that the evoked PC firing for temporal control of movement is encoded in the cerebellum by beams of synchronously active PCs (Jaeger, 2003). So, in our model, PC responses were driven by 1,000 synchronized PF inputs randomly distributed on spiny dendrites (Su et al., 2012; Hoxha et al., 2016), i.e., simulations were run with a synchronized stimulation protocol.

A single stimulation consists of a sequence of spikes containing spike times and inter-spike intervals (ISIs), so we can generate a successive spike train by the previous spike plus the regular or irregular time intervals. There are three types of stimulations used in this study depending on the sampling process.

#### 2.3.1. Poisson Process

Spike trains were modeled using a homogeneous Poisson process in which the ISI distribution is exponential. The probability density function of ISI τ is given by:

(7) $$\begin{array}{l} p\left(\tau \right)=re^{-r\tau } \end{array}$$

where *r* is mean firing rate. The mean < τ > and standard deviation σ of ISIs are both 1/*r*.

#### 2.3.2. Renewal Process

A simple way to generate a spike train based on the renewal process is to start with a Poisson spike train and delete all but every *k*-th spike. In this way, a spike train is obtained with ISIs τ given by gamma probability density function:

(8) $$\begin{array}{l} p\left(\tau \right)={\left(k\tau \right)}^{k}\tau^{k-1}e^{-kr\tau }/\left(k-1\right)! \end{array}$$

where *k* is the order of gamma distribution, and *r* is the mean firing rate. The mean < τ > and standard deviation σ are 1/*r* and$<\tau >/\sqrt{k}$, respectively. Here k is set to 2 throughout the whole study.

#### 2.3.3. Modulated Renewal Process

Recent work on the statistical modeling of neural responses has focused on modulated renewal processes in which the spike rate is a function of the recent spiking history. Here we modeled the modulation of the firing rate *r*(*t*) as a synaptic input frequency such that

(9) $$\begin{array}{l} r\left(t\right)=Asin\left(2\pi ft+\theta \right)+A \end{array}$$

where the modulation of the firing rate is thus fully specified by its amplitude *A* and frequency *f* of the sinusoidal component. Thus, one can generate a spike train that satisfies this firing rate *r*(*t*) by the time-rescaling method (Brown et al., 2002; Pillow, 2009; Zampini et al., 2016).

### 2.4. Data Analysis

The full and reduced PC morphology models were stimulated in NEURON 7.4. Data analysis was implemented with MATLAB. Unless otherwise noted, all simulations ran for 2,000 ms. where data from the last 1,000 ms simulation were used to analyze the results. The time step 0.025 ms was used for all simulations. For analysis, we used four measures to characterize the performance of the reduced method: accuracy, simplification, efficiency and spike shape change.

#### 2.4.1. Accuracy

The main purpose of a reduced model is to maintain the input-output (I/O) property on the basis of simplification of neuronal morphology. We evaluated the accuracy of the I/O property by comparing the spike trains generated before and after reduction (Marasco et al., 2013):

(10) $$\begin{array}{l} \text{Accuracy}=\frac{\text{TP}+\text{TN}}{\text{TP}+\text{TN}+\text{FP}+\text{FN}} \end{array}$$

where TP (True Positives) is the number of spikes from the full model that are also found in the reduced model. TN (True Negatives) is the number of intervals that the neuron does not fire in both the full and reduced models. FP (False Positives) is the number of mismatched spikes in the reduced models. FN (False Negatives) is the number of spikes from the full model that are not matched in the reduced models.

#### 2.4.2. Simplification

Morphological simplification is the most basic requirement for a reduced method. The full morphological structure can be seen as being made up by a series of cylindrical segments with different lengths and diameters. Therefore, we characterized the degree of simplification as the ratio of the segments of morphological structure before and after reduction:

(11) $$\begin{array}{l} \text{Simplification}=\frac{\text{SE}{\text{G}}_{\text{full}}-\text{SE}{\text{G}}_{\text{reduced}}}{\text{SE}{\text{G}}_{\text{full}}} \end{array}$$

where SEG_reduced_ is the number of the segments in the reduced morphological structure, and SEG_full_ is the number of the segments in the full morphological structure.

#### 2.4.3. Efficiency

Another feature of a reduced model is to improve computing efficiency, as one wants to compute the neuronal dynamics as fast as one can, in particular in large-scale network simulations. We evaluated this feature by the ratio between the runtime of the full model and reduced model in the same computing environment:

(12) $$\begin{array}{l} \text{Efficiency}=\frac{\text{Runtim}{\text{e}}_{\text{full}}}{\text{Runtim}{\text{e}}_{\text{reduce}}} \end{array}$$

where Runtime_full_ is the run time of the full model and Runtime_reduce_ is the run time of the reduced model.

#### 2.4.4. Spike Shape

In addition, we also analyzed the accuracy of spike shapes including spike width and spike amplitude. We evaluated this feature by comparing the changes in the full model and the reduced model.

(13) $$\begin{array}{l} \text{Δ}Chang{\text{e}}_{X}=|Full_{X}-Reduc{\text{e}}_{X}| \end{array}$$

Where X represents spike width or spike amplitude, *Full*_*X*_ and *Reduce*_*X*_ are the mean spike width or spike amplitude in spike trains of the full model and reduced model, respectively.

## 3. Results

We considered four different reduction schemes to simplify the morphology of PCs from three species, then studied their effects on PC responses to synaptic inputs. To investigate how the coding capacity of PCs is affected by morphological structure reductions, we evaluated the performance of the reduced model with four measures: simplification, accuracy, efficiency, and spike shape change. Furthermore, the coding capacity of PCs, in terms of firing rate coding, spike timing coding, and modulated firing amplitude and phase under different input synaptic frequencies were studied as well.

### 3.1. Performance Evaluation of Morphology Reduction

**Figure 2:**
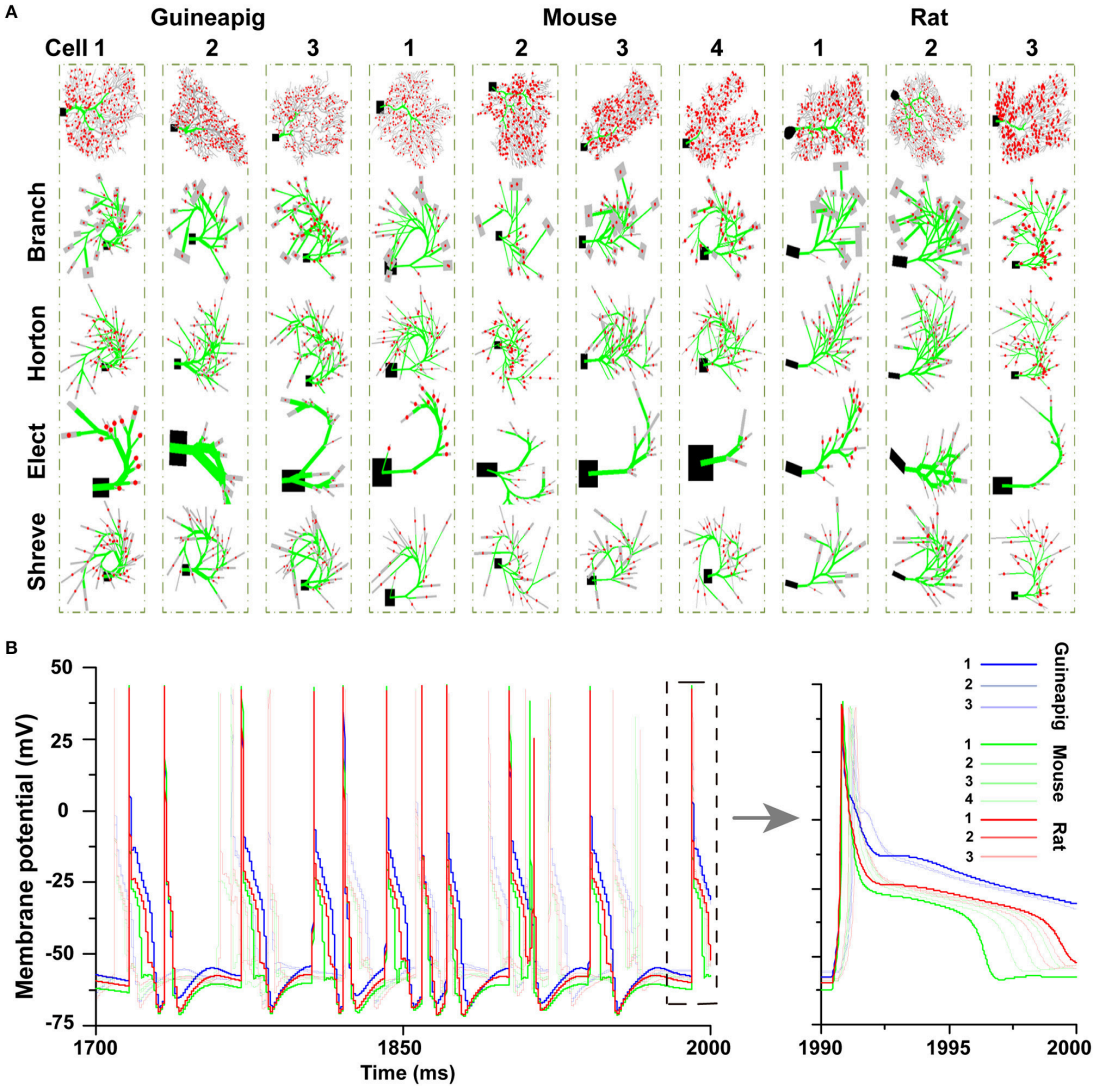
Detailed and simplified PC morphologies. **(A)** A total of 10 PCs from three species are reduced by four different schemes, Branch, Horton, Elect, and Shreve methods. Spiny dendrites in gray, smooth dendrites and initial major branches in green. Spiny dendrites receive 1,000 excitatory AMPA-type synapses from parallel fibers (red dots). **(B)** Hyperpolarization phases after spiking are different in 10 full PC morphologies. Poisson stimulation at 50 Hz.

Ten different PCs with different morphologies from guinea-pigs, mice, and rats were studied. The reduced morphologies were generated by four types of reduction schemes for each cell as shown in Figure 2A. Four reducing schemes show different simplifications for a particular morphology. However, there is a large variety of neuronal morphology for each cell from each species. Although the same set of parameters were used in all 10 PCs, different morphologies resulted in different dynamics of hyperpolarization. Such a difference is larger across species and smaller within the same species (Figure 2B).

**Figure 3:**
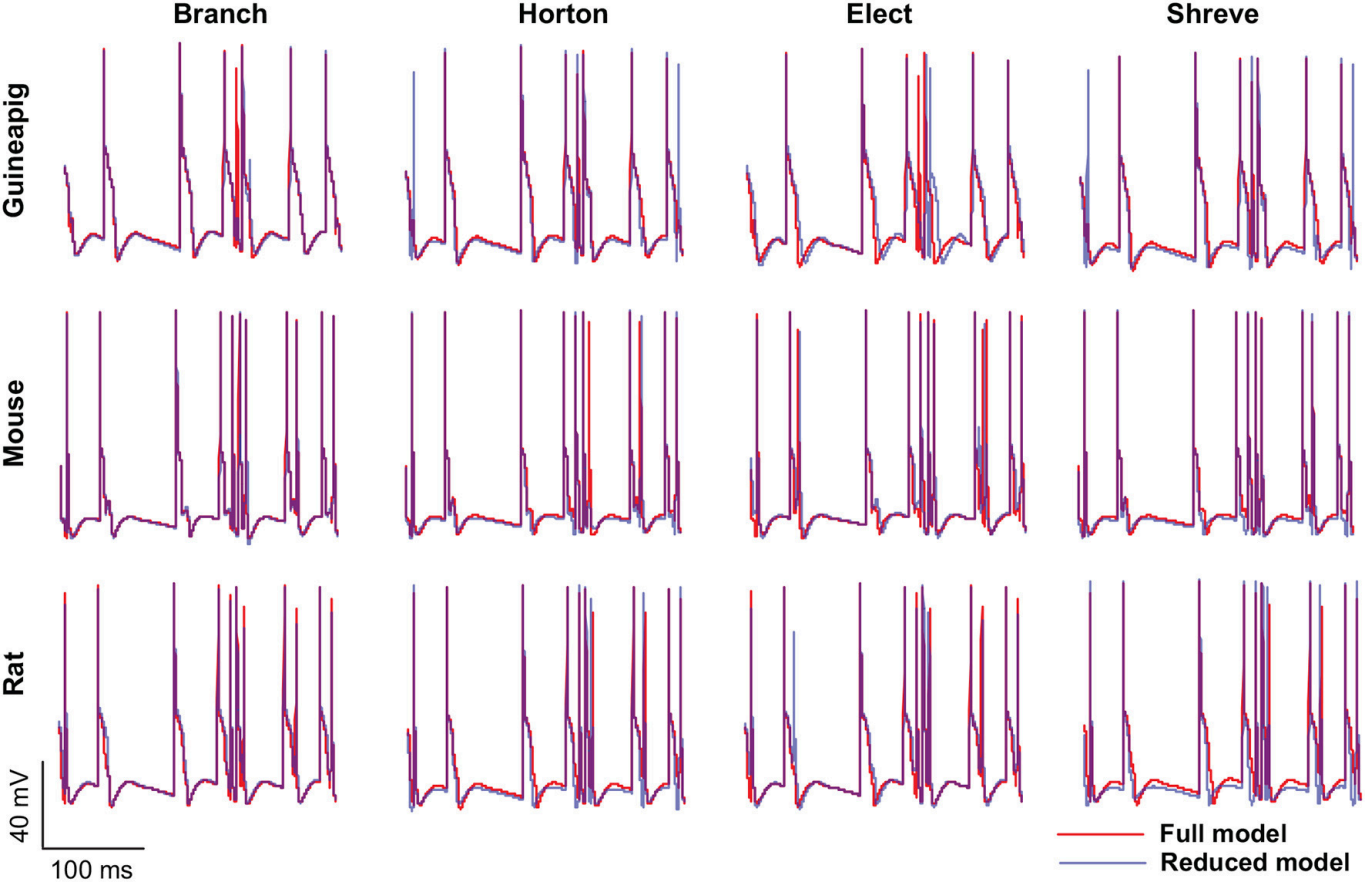
Spiking dynamics of full and reduced PC models. Membrane potential traces recorded from the soma of three example PCs of guinea-pigs, mice and rats with full model (red) and four reduced models (blue). Poisson stimulation at 50 Hz.

Then, we focused on three example PCs, guinea-pig1 (v_e_purk1Mod), mouse1 (e4cb2a2Mod), and rat1 (p20) to illustrate the spiking dynamics of full and reduced morphology. Throughout the study, these three example PCs are used to represent three species respectively. In general, for all PCs, the spiking dynamics of reduced morphology in all four schemes matched that of the full model very well (Figure 3).

It is especially important to ensure the firing accuracy of the reduced model, which can be seen in Figure 3. The detailed measure quantified by *accuracy* shown in Table 2, which describes how a somatic membrane potential trace of the full model can be reproduced in different reduced models. For comparison, Marosco model was included (Marasco et al., 2013). The accuracy is relatively diverse in four reduced schemes, but in general, the Branch model has the highest accuracy. However, the Marosco reduced model does not archive accurate firing activities.

**Table 2:** Accuracy of reduced models.

	**Guinea-pig1**	**Guinea-pig2**	**Guinea-pig3**	**Mouse1**	**Mouse2**	**Mouse3**	**Mouse4**	**Rat1**	**Rat2**	**Rat3**
Branch	**0.976 ± 0.012**	0.940 ± 0.031	**0.973 ± 0.017**	0.926 ± 0.057	0.905 ± 0.063	**0.984 ± 0.013**	**0.965 ± 0.019**	0.941 ± 0.036	**0.963 ± 0.024**	**0.964 ± 0.029**
Horton	0.954 ± 0.021	**0.942 ± 0.029**	0.971 ± 0.015	0.860 ± 0.078	0.885 ± 0.064	0.949 ± 0.032	0.880 ± 0.069	0.934 ± 0.038	0.926 ± 0.035	0.955 ± 0.028
Elect	0.886 ± 0.021	0.825 ± 0.048	0.880 ± 0.040	**0.970 ± 0.019**	0.860 ± 0.063	0.938 ± 0.029	0.848 ± 0.047	**0.968 ± 0.015**	0.781 ± 0.074	0.850 ± 0.052
Shreve	0.943 ± 0.023	0.924 ± 0.035	0.972 ± 0.012	0.906 ± 0.054	**0.913 ± 0.052**	0.959 ± 0.020	0.939 ± 0.033	0.889 ± 0.064	0.954 ± 0.017	0.921 ± 0.050
Marosco	0.932 ± 0.029	0.929 ± 0.029	0.930 ± 0.027	0.872 ± 0.092	0.881 ± 0.059	0.955 ± 0.022	0.939 ± 0.033	0.906 ± 0.051	0.947 ± 0.019	0.908 ± 0.057

*Values are mean ± STD calculated from 21 sets of Poisson stimulation from 10 to 1K Hz. The best reduced method for each cell is marked in bold*.

The second measure is *simplification* that characterizes how the morphologies are changed by reduced models. The reduced model simplifies complex tree structures into a much smaller set of dendrites that can be quantified by the degree of simplification. Table 3 shows that the Elect model has the largest degree of simplification such that the reduced morphology is most compact. In contrast, the Horton model has a minimum degree of simplification. The Marosco model has a slightly larger degree of simplification than the Elect model for mouse2 and rat1.

**Table 3:** Simplification of reduced models.

	**Guinea-pig1(%)**	**Guinea-pig2(%)**	**Guinea-pig3(%)**	**Mouse1(%)**	**Mouse2(%)**	**Mouse3(%)**	**Mouse4(%)**	**Rat1(%)**	**Rat2(%)**	**Rat3(%)**
Branch	85.5	93.2	80.1	93.3	90.3	86.0	85.7	86.6	90.7	82.8
Horton	79.5	82.1	79.5	79.7	77.2	78.5	78.8	81.8	83.6	80.6
Elect	**96.7**	**96.9**	**96.2**	**95.7**	91.9	**97.1**	**98.9**	93.1	**94.3**	**97.7**
Shreve	88.8	90.7	87.6	91.7	88.8	89.7	88.5	93.3	91.7	91.0
Marosco	91.4	93.0	95.1	93.9	**92.2**	92.7	95.1	**94.6**	94.1	93.9

*The best reduced method for each cell is marked in bold*.

A direct outcome of simplification is that the simplest model needs less computing time for simulation. It is feasible to ensure the application of reduced models on large-scale networks only by improving computational efficiency while high accuracy is maintained. Table 4 shows the quantification of *runtime*, where the Elect model achieves the most efficient computation, as it has the highest degree of simplification, except that the Marosco model is fastest for rat1.

**Table 4:** Efficiency of reduced models. Values are mean ± STD.

	**Guinea-pig1**	**Guinea-pig2**	**Guinea-pig3**	**Mouse1**	**Mouse2**	**Mouse3**	**Mouse4**	**Rat1**	**Rat2**	**Rat3**
Branch	19 ± 3	22 ± 8	9 ± 2	24 ± 7	20 ± 2	15 ± 2	15 ± 3	13 ± 1	21 ± 6	12 ± 3
Horton	9 ± 2	9 ± 2	8 ± 1	11 ± 1	9 ± 1	9 ± 1	10 ± 2	11 ± 2	13 ± 2	10 ± 2
Elect	**40 ± 8**	**59 ± 12**	**46 ± 8**	**56 ± 9**	**30 ± 4**	**64 ± 6**	**72 ± 1**	16 ± 1	**39 ± 13**	**41 ± 2**
Shreve	16 ± 3	18 ± 3	12 ± 1	18 ± 3	13 ± 1	14 ± 1	14 ± 2	20 ± 2	17 ± 5	16 ± 4
Marosco	24 ± 6	27 ± 4	35 ± 4	31 ± 3	23 ± 2	24 ± 3	26 ± 1	**22 ± 1**	24 ± 4	22 ± 3

*The best reduced method for each cell is marked in bold*.

Furthermore, it is also important to maintain the accuracy of spike shape, which can be measured by the change of spike amplitude in Table 5 and spike width in Table 6, where the Branch model has higher accuracy for more cells.

**Table 5:** Change of spike amplitude in reduced models (mv, mean ± STD).

	**Guinea-pig1**	**Guinea-pig2**	**Guinea-pig3**	**Mouse1**	**Mouse2**	**Mouse3**	**Mouse4**	**Rat1**	**Rat2**	**Rat3**
Branch	**1.08 ± 0.53**	3.63 ± 1.95	1.63 ± 0.98	0.68 ± 0.88	6.23 ± 2.74	**0.48 ± 0.17**	1.03 ± 0.57	3.71 ± 1.82	3.87 ± 1.87	**0.78 ± 0.27**
Horton	2.08 ± 1.02	3.12 ± 1.37	1.42 ± 0.62	1.03 ± 0.24	7.30 ± 3.38	0.63 ± 0.16	**0.55 ± 0.58**	1.81 ± 0.55	0.62 ± 0.27	0.91 ± 0.28
Elect	1.58 ± 0.80	**3.09 ± 1.36**	**1.24 ± 1.00**	**0.54 ± 0.22**	12.10 ± 5.89	2.00 ± 0.74	5.17 ± 3.30	**0.95 ± 0.50**	2.85 ± 0.70	1.72 ± 1.43
Shreve	3.02 ± 1.40	4.98 ± 2.60	1.95 ± 1.02	0.86 ± 0.19	**4.95 ± 2.49**	0.81 ± 0.26	0.89 ± 0.65	3.56 ± 1.32	**0.56 ± 0.18**	1.98 ± 0.53
Marosco	3.86 ± 1.88	4.40 ± 2.18	4.00 ± 1.83	1.80 ± 0.48	6.61 ± 2.89	1.04 ± 0.30	0.80 ± 0.61	3.81 ± 1.34	0.79 ± 0.24	2.55 ± 1.24

*The best reduced method for each cell is marked in bold*.

**Table 6:** Change of spike width in reduced models (ms, mean ± STD).

	**Guinea-pig1**	**Guinea-pig2**	**Guinea-pig3**	**Mouse1**	**Mouse2**	**Mouse3**	**Mouse4**	**Rat1**	**Rat2**	**Rat3**
Branch	**0.29 ± 0.23**	0.73 ± 0.29	**0.18 ± 0.16**	0.20 ± 0.25	1.02 ± 0.88	**0.06 ± 0.08**	0.53 ± 0.50	0.28 ± 0.26	0.92 ± 1.07	**0.09 ± 0.09**
Horton	0.34 ± 0.27	**0.41 ± 0.32**	0.23 ± 0.15	**0.20 ± 0.18**	0.84 ± 0.44	0.25 ± 0.27	0.81 ± 0.32	0.18 ± 0.19	0.30 ± 0.19	0.13 ± 0.18
Elect	0.81 ± 0.62	1.39 ± 1.78	0.44 ± 0.45	0.26 ± 0.14	**0.51 ± 0.74**	0.34 ± 0.21	0.4 ± 0.4	**0.11 ± 0.12**	0.30 ± 0.23	0.49 ± 0.19
Shreve	0.42 ± 0.39	0.59 ± 0.42	0.27 ± 0.35	0.23 ± 0.14	0.92 ± 0.45	0.21 ± 0.15	0.55 ± 0.32	**0.37 ± 0.37**	0.28 ± 0.22	0.36 ± 0.43
fMarosco	0.49 ± 0.33	0.52 ± 0.33	0.41 ± 0.32	0.31 ± 0.16	0.57 ± 0.41	0.18 ± 0.23	**0.31 ± 0.13**	0.34 ± 0.46	0.32 ± 0.22	0.47 ± 0.69

*The best reduced method for each cell is marked in bold*.

Given that accuracy is the most important factor for reduction, the Branch model is the best one for the performance of the accuracy of the spike rate, the degree of simplification, and the runtime and spike shape, are also good. The reason for this is mainly due to the fact that the scaling factor in the simplification process preserves the cell volume in the Branch model, while the other three models preserve the cell membrane area. Thus, we conclude that cell volume may play an important role in shaping Purkinje cell firing.

### 3.2. PC Firing Rate Coding

**Figure 4:**
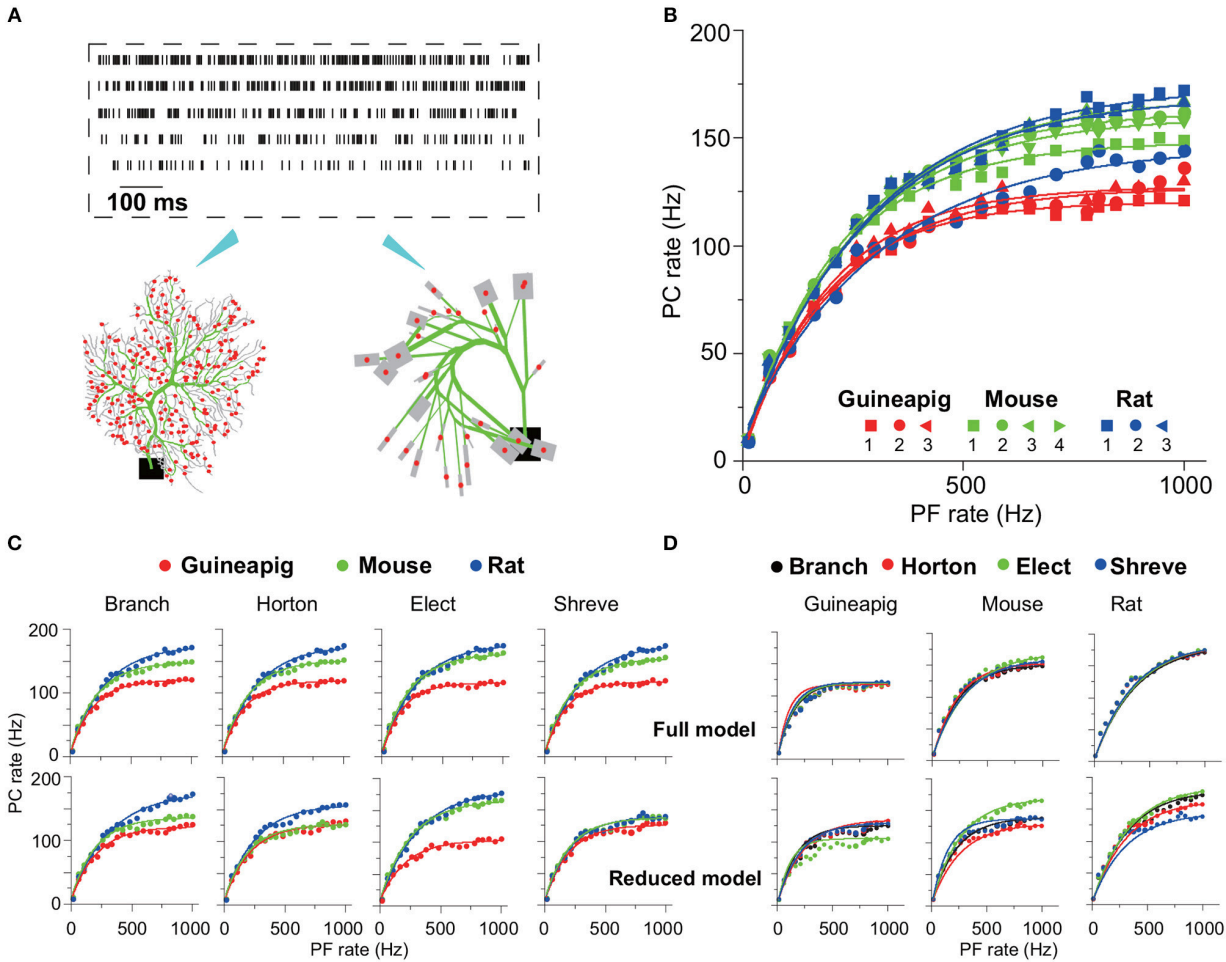
PC firing responses in full and reduced models. **(A)** Schematic view of Poisson stimulation sequences from 60 to 260 Hz injected to spiny dendrites of full (left) and reduced (right) models. **(B)** Firing response curves of 10 PCs with full morphology. **(C,D)** Comparison of firing response curves of three example PCs from a guinea-pig, mouse and rat under four reduction schemes, Branch, Horton, Elect, and Shreve, respectively. PC curves are grouped by species in (C) and by reduction schemes in **(D)**. Note that there are four PC response curves in one full model, since each is a realization of random distribution of PF input synapses. Solid curves in **(B–D)** are fitted exponential functions. Poisson stimulation in all cases.

To characterize the performance of reduced models, we carried out a wide range of stimulation frequencies from 10Hz to 1KHz sampled in the Poisson process as illustrated in Figure 4A, which is in the same range as observed *in vivo* experiments where granule cells can discharge from a few Hz up to 1K Hz (Valera et al., 2012).

The cerebellum can control high-precise motor patterns with millisecond resolution using a wide range of action potential firing rates (Amir and Zee, 2011; Ostojic et al., 2015; Jelitai et al., 2016). Figure 4B shows 10 PC firing rate response curves under Poisson stimuli ranging from 10 Hz to 1K Hz in the full morphology model. When such stimuli are used, different species have their own response frequency range, except for PC rat2 that has a lower range compared to the other two rat PCs.

There is a variation of PC response curves within the same species due to their morphological differences. The PC firing rates dramatically increase with stimulations from 10 to 180 Hz, and then slowly saturate at the higher stimulation frequencies, which is similar to the experimental observations found in awake animals, where PCs exhibit an action potential rate between 30 and 200 Hz (Bryant et al., 2010; Cao et al., 2017).

By using the same three example PCs as in Figure 3, one can compare the detailed differences of PC response across different species and reduced models. Figure 4B shows that the guinea-pig PC response is significantly lower than the mouse and rat, in particular, during high-frequency stimulation. This is consistent with the difference shown in the phase of hyperpolarization after spiking (Figure 2 B), even other parameters were fixed as the same. This may imply that rats and mice are able to react on millisecond timescale activities faster than guinea-pigs. Four different reduced methods divide the PCs into different spiny dendrites, which leads to synaptic distribution locations slightly different, as a result, four corresponding full models are slightly different in their response curves (Figures 4C,D). The response frequency has to be high enough when the stimulation frequency is high, as evidenced by experimental data that PC could generate a high stage firing rate to control fast timing patterns of the motor behavior. PC response curves can be well fitted by exponential function although the high frequency slowly saturates. After morphology is reduced, PC response curves are preserved from the full model, but there are some differences depending on the reduction schemes as shown in Figures 4C,D. Shreve and Horton models mediate PC firing rate decreased compared to the full model at high stimulation frequencies. This could be seen from both the mouse and rat, but the guinea-pig showed a slightly increased PC firing rate. In the Elect model, the guinea-pig showed a significant decrease in firing rate. In the Branch model, the PC rate decreased in the mouse while the guinea-pig and rat showed similar firing rates. The increased diversity at high frequency stimulation implies that complex spatial morphology structures can affect PCs to react to millisecond-scale activity. In general, the Branch model is the best model to preserve the input-output relationship of PCs in all species.

Similar results were also observed when the stimulation protocol was changed from Poisson to renewal process (Supplementary Figure 1). Therefore, PC firing responses vary depending on morphology and species rather than the types of stimulation used.

### 3.3. PC Timing Coding

Purkinje cells transmit precise timing information to their downstream targets for precise control of motor-related tasks and conditioned behaviors (Koekkoek et al., 2003; Ivry and Spencer, 2004). Here, by using the full and reduced models with different stimulation protocols, we investigated the effect of morphology structures on PC temporal coding, in particular, we focused on simple spikes that are the majority of PC spikes quantified by their inter spike intervals (ISIs), which has shown that statistics of ISIs play an important role in PC temporal coding (Shin and Schutter, 2006; Shin et al., 2007).

**Figure 5:**
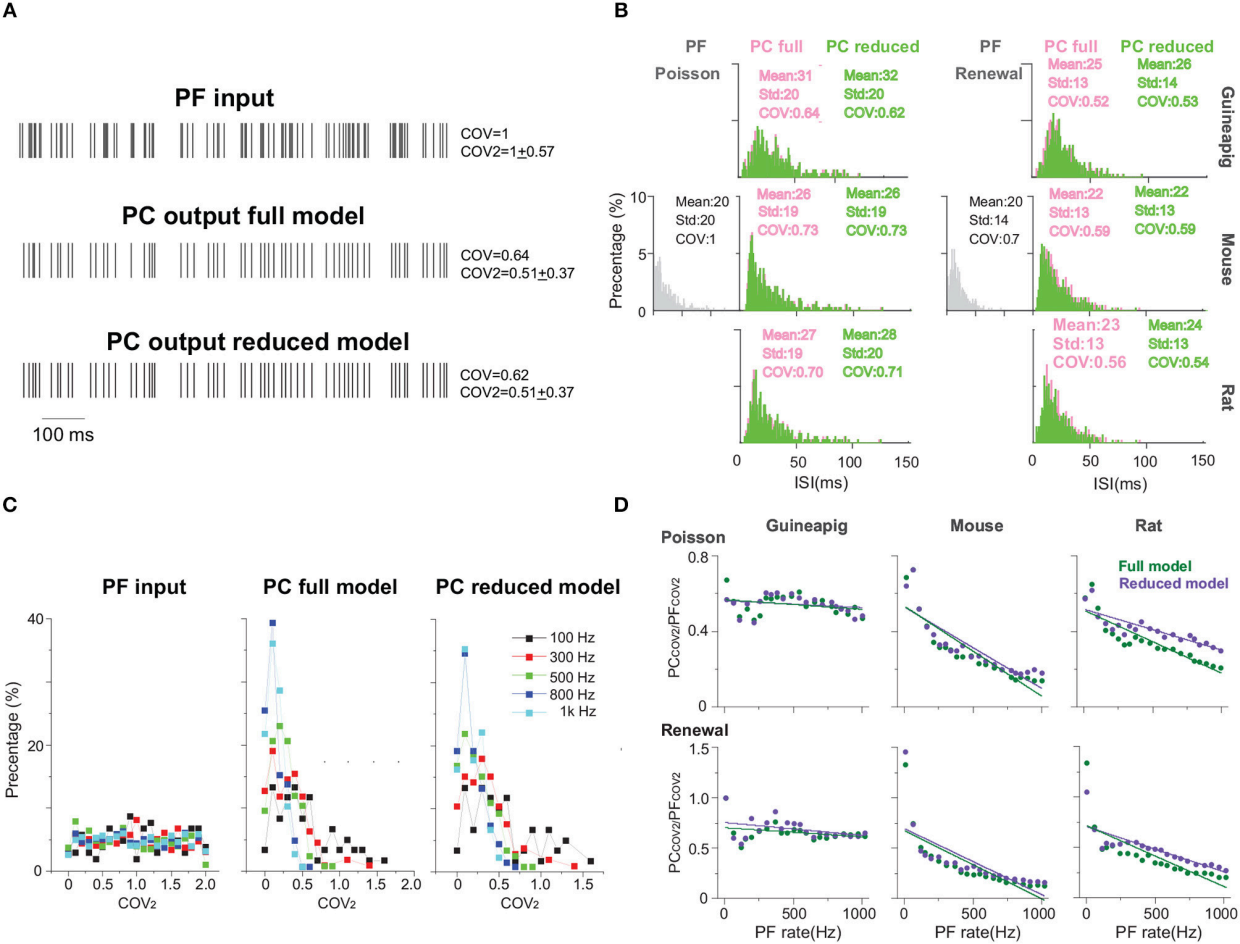
PC temporal responses under Poisson and renewal stimulation with Branch method. **(A)** Schematic view of PF input (top) and PC output spike trains from full model (middle) and Branch model (bottom) from mouse. Poisson stimulation at 100 Hz. **(B)** ISI distribution of spike trains from PF (gray), PC full model (light red) and PC reduced model (green), respectively, under Poisson and renewal process stimulation for guinea-pig (top), mouse (middle), rat (bottom). All stimuli are at 50 Hz for 10 s. Similarity between the ISI distributions of full and reduced model measured by *p*-value, Wilcoxon Rank-sum test. Guinea-pig, 0.51; Mouse, 0.97; Rat 0.26 for Poisson stimulation, and Guinea-pig, 0.63; Mouse 0.93; Rat, 0.32 for renewal stimulation. **(C)** Distribution of COV_2_ values obtained from spike trains of PF inputs (left), PC full model (middle), and Branch model (right) from mouse with Poisson stimulation of different frequencies. **(D)** PC_COV2_/PF_COV2_ showing the regularity between PF inputs and PC outputs for full model (green) and reduced model (purple) of guinea-pig, mouse and rat. Poisson and renewal process stimulation with different frequencies from 10 to 1K Hz.

Figure 5 shows the results of temporal coding precision of three of the same PCs from a guinea-pig, mouse, and rat, under the stimulation protocols of the Poisson and renewal processes. A schematic view of PF inputs and PC outputs of the full and reduced models illustrates that the Branch model reduces the full morphology but keeps the temporal coding very accurately (Figure 5A). A common way to characterize the temporal structure of the spike train is to use the coefficient of variation (COV) of ISIs. Based on this measure, the spike trains generated by the PCs are significantly more regular than those of PFs. The detailed statistics of ISIs of three example PCs with both full and reduced models confirm this observation (Figure 5B). Furthermore, the ISI distributions obtained by the reduced model are similar to those by the full model, which can be described by *p*-values from the Wilcoxon Rank-sum test. For all three PCs and both stimulation protocols, *p*-values of two distributions of the full and reduced model are non-significant. For the same frequency stimulus, the PC ISI distributions are different from each other across the two stimulation protocols of Poisson and the renewal process, and also different across species as well.

Fine characterization of temporal precision of spike trains can be described by a modified measure COV_2_ = 2|ISI_n+1_ − ISI_n_|/(ISI_n+1_ + ISI_n_), which can avoid that one of the ISI in the sequence affects global regularity (Shin et al., 2007; Hong et al., 2016). A single spike train can obtain a sequence of COV_2_ values (Supplementary Figure 3), and their distributions arle spike train can obtain a sequence ofe shown in Figure 5C for several example frequencies of Poisson stimulation. Not surprisingly, the COV_2_ values of the PF stimulation sequence are distributed uniformly with different Poisson frequencies. However, the COV_2_ values of PC spike trains have a wide distribution with the peak around 0.1.

One can average all COV_2_ values for each spike train, then use the value of the ratio PC_COV_2__/PF_COV_2__ as an indicator to evaluate the regularity between PF inputs and PC outputs, as shown in Figure 5D, such that the ratio >1 indicates that PF firing is more regular, otherwise, PC is more regular. Similar to ISI distribution, there are clear differences across species. Changing of firing regularity was found in the mouse and rat, but not in the guinea-pig. It is worth noting that PC spike trains are more regular with the only exception that the ratio is close to 1 for the renewal process in a low stimulus frequency (10 Hz) (Supplementary Figures 2D–F), while PC spike trains are always more regular for the Poisson stimulation.

Furthermore, the performance of different reduced models to capture the temporal structure of spike trains shown in the full model is different. Under the same stimulation condition, the spike trains of the mouse PC show more regularity in the Horton and Shreve models, but the rat shows more regularity in the Branch and Shreve models. However, the guinea-pig only shows more regularity in the Elect model under the renewal process stimulation. In addition, both full and reduced models have similar results under different stimulations protocols (Supplementary Figure 2).

Cerebellar PCs are observed to generate regular spike trains *in vivo* (Häusser and Clark, 1997; Hong et al., 2016). PC simple spike trains contain highly regular spiking patterns and may transfer information coded by regular spike patterns to downstream deep cerebellar nuclei neurons (Shin et al., 2007).

**Figure 6:**
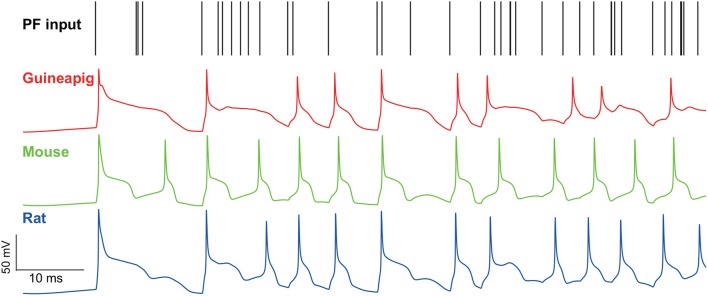
Membrane potential traces recorded from the soma of three example PCs of a guinea-pig, mouse and rat in the Branch model. Poisson stimulation at 500 Hz.

To see this effect, we applied a wide range of stimulation frequencies to extract the regular and irregular spiking patterns. A zoomed-in illustration of firing activity is shown in Figure 6 under 500 Hz Poisson stimulation, which shows that the guinea-pig has the most irregular refractory periods as demonstrated in Figure 2 where there is a much slower hyperpolarization phase for the guinea-pig, compared to the mouse and rat under the same stimulus condition.

**Figure 7:**
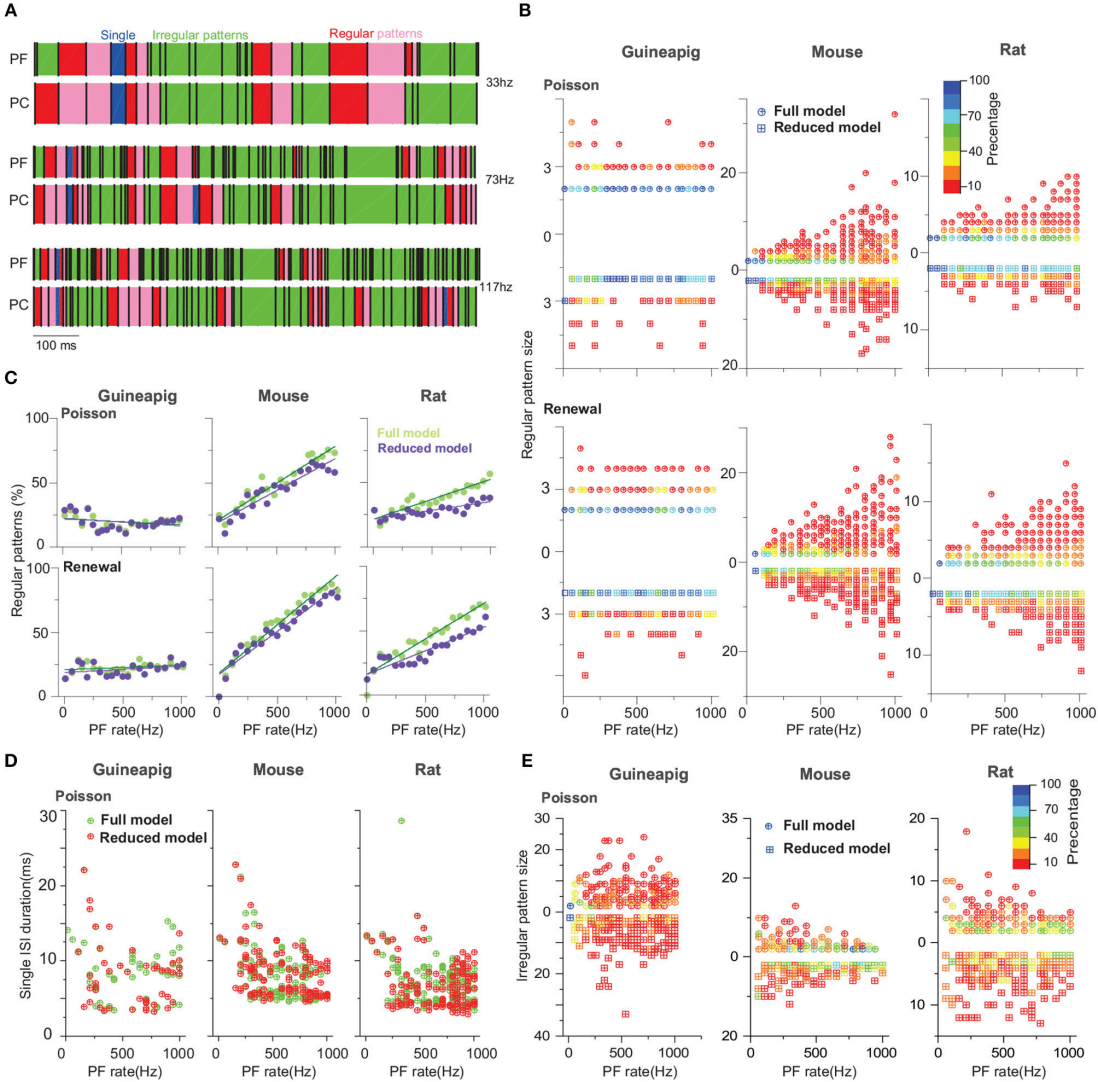
PC spiking pattern statistics with Branch method. **(A)** Illustration of regular and irregular spiking patterns from spike trains. Each black bar indicates a spike. The start of regular patterns is colored in red; pink indicates successive ISIs in regular patterns, an irregular pattern is in green, and single ISI is in blue. Mouse PC was used. **(B)** Statistics of regular pattern size across a range of Poisson and renewal process stimulation for a guinea-pig, mouse, and rat in both the full and reduced models. Percentage of different size indicated by different colors, as there are more patterns in a higher frequency. **(C)** Percentage of regular patterns at different stimulation frequencies. **(D)** Single ISI duration across a range of a Poisson stimulation. **(E)** Statistics of irregular pattern size across a range of Poisson stimulation. Percentage of different size indicated by different colors.

To characterize this in detail, we applied a threshold of 0.2 on the measured COV_2_ values similar to the previous study (Shin et al., 2007) to extract a series of segments of regular spiking patterns in individual spike trains obtained by the Branch model as illustrated in Figure 7A.

Not surprising, when stimulus frequency is larger, regular patterns have small ISIs due to increased noise (Supplementary Figure 4). However, the statistics of regular spiking patterns across species are quite different as shown in Figure 7C. Increasing of input frequency results in more regular patterns for the mouse and rat, but there is no significant change for the guinea-pig. Results of all four reduced models are similar to the full model for the guinea-pig. For the mouse, the difference is significantly larger in the Horton and Shreve models, whereas this difference is very weak in the Branch and Elect models. The rat PC shows more regular patterns in the full model than the Branch and Shreve reduced models, but this difference is not obvious in the Horton model (see Supplementary Figure 7).

The size of the regular pattern is defined as the number of ISIs in regular patterns. Most of the patterns have only 2-3 ISIs for guinea-pig. Interestingly, for the mouse and rat the regular pattern sizes are widely distributed at high frequencies, where there are quite a few longer regular patterns in the mouse as shown in Figure 7B and Supplementary Figure 8.

Figure 7D shows that the guinea-pig has fewer single ISI and the single ISI duration distribution is more discrete at low frequencies Supplementary Figure 5A. In contrast to the regular pattern, irregular pattern sizes are widely distributed at low frequencies for the mouse and rat, however, the guinea-pig has a larger irregular size as shown in Figure 7E and Supplementary Figures 5B, 9.

Thus, we conclude that reduced models can capture the PC temporal coding in a reasonable range, which is in particular important for maintaining precise timing patterns converted by PCs to theirs downstream deep cerebellar nuclei neurons, to control motor patterns.

### 3.4. PC Coding of Modulated Inputs

In the previous sections, we demonstrated the input-output relationships of PC firing activity by Poisson and renewal process stimulations with a constant firing rate. Recently, *in vivo* studies show that the modulations of PC simple spike firing are related to a number of functions in terms of behavior, prediction, and sensory feedback (Cao et al., 2012; Streng et al., 2018). In order to elucidate a simple spike firing modulation, we simulated the dynamics of the PC in response to PF inputs with modulated firing amplitude and frequency. As the activities of PFs from granular cells are represented by modulations of stimuli, one can simply model PF inputs as sinusoidal modulations (Zampini et al., 2016) that can generate PF spike trains from a modulated renewal process. By varying the amplitude and frequency of sinusoidal input, we investigated the ability of firing modulation of PC simple spikes under different reduced models.

**Figure 8:**
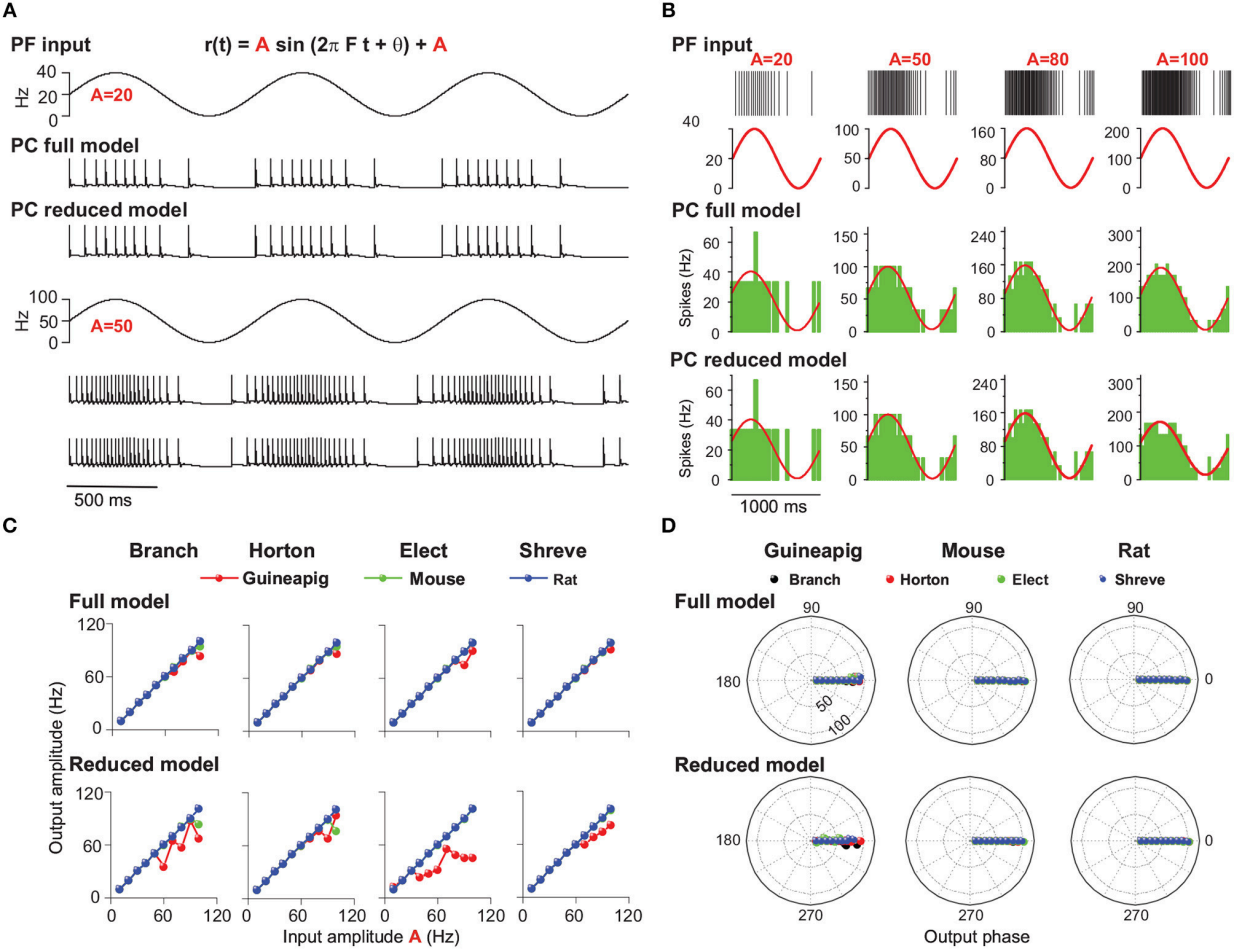
PC firing modulated by sinusoidal PF inputs with different amplitudes. **(A)** Illustration of PF inputs and PC outputs. PF sinusoidal input at 1 Hz frequency with amplitude 20 (top) and 50 (bottom). Modulated PC firing voltage traces over three cycles of input for full model and Branch model. **(B)** Similar to **(A)** with four different amplitudes of 20, 50, 80, and 100 Hz inputs. (top) PF input spike trains and modulated firing rates. PC spike responses from the (middle) full model and (bottom) Branch model. Mouse PC used in **(A,B)**. **(C)** Comparison of modulation amplitudes of PF input vs. PC output in full and reduced models of four reduced schemes for the guinea-pig (red), mouse (green), and rat (blue), respectively. **(D)** Similar to **(C)**, but for phase change of PC firing modulation for full model and reduced models. Sinusoidal stimulation frequency is 1Hz in all cases.

We first set out to analyze PC firing modulation by changing PF input amplitude, where frequency and phase of sinusoidal input is 1 Hz and 0, respectively. Figure 8A shows example results of mouse PC firing in response to modulated PF inputs at two different amplitudes, together with responses from the reduced Branch model. The amplitude of PC modulation is expected to be equal to that of the oscillating input when the reduced model is accurate enough, which is shown in Figure 8B with four tightly matched example responses for the mouse PC.

For the full model, the modulated amplitudes of the mouse and rat PC responses are well matched to those of PF inputs (Figure 8C). However, the guinea-pig PC is different: well-matching is observed for lower amplitudes up to 90 Hz (80 Hz in Elect method), but smaller than PF input amplitude for larger input amplitudes. Similarly, the PC firing of the guinea-pig shows in phase with low amplitude PF inputs, but slightly leads phase at high input amplitude (large than 100 Hz). However, the mouse and rat PC modulation phases are in phase with PF inputs for all input frequencies (Figure 8D).

To investigate the firing modulation when PC morphological structures are changed, we simulated four corresponding reduced models. Compared to the full model, the guinea-pig shows lower modulation amplitudes for all reduced models. The mouse shows lower modulation amplitudes only in the Branch and Horton reduced models with higher sinusoidal amplitude (90 Hz). However, the modulation amplitudes of the rat are always matched to the PF input amplitudes in the four reduced models (Figure 8C). Moreover, in four reduced models, the modulation phases of the mouse and rat are always in phase with PF inputs. However, the guinea-pig shows leading phases at low input amplitudes and lagged phases at high input amplitudes. Therefore, when the modulation amplitude changes, there is a large influence on PC modulated amplitudes and little influence on the PC modulated phase. In particular, for the guinea-pig, the complex tree structure plays a key role in the firing modulation.

**Figure 9:**
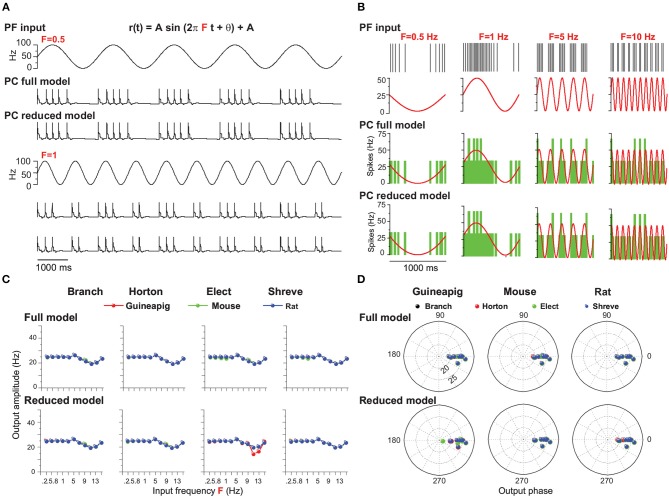
PC firing modulated by sinusoidal PF inputs with different frequencies. **(A)** Illustration of PF inputs and PC outputs. PF sinusoidal input with amplitude 50 at frequencies of 0.5 Hz (top) and 1 (bottom). Modulated PC firing voltage traces over five cycles of input for full model and Branch model. **(B)** Similar to **(A)** with four different frequencies of 0.5, 1, 5, and 10 Hz inputs. (top) PF input spike trains and modulated firing rate. PC spike responses from the (middle) full model and (bottom) Branch model. Mouse PC used in **(A,B)**. **(C)** Modulation amplitudes of PC output in full and reduced models of four reduced schemes for the guinea-pig (red), mouse (green), and rat (blue), respectively, at different PF input frequencies. **(D)** Similar to **(C)**, but for phase change of PC firing modulation for the full and reduced models. PF sinusoidal stimulation amplitude is 25 Hz in **(B–D)**.

Recently, theoretical studies showed the rate of PC tonic firing could be modulated by somatic injection of sinusoidal currents up to remarkably high frequency (1 kHz) (Ostojic et al., 2015). Here we replaced the current input with the modulated synaptic input as above, changed the frequency of PF sinusoidal input, and analyzed its effect on PC firing modulation as shown in Figure 9.

Figure 9A shows example results of mouse PC firing modulation with given PF sinusoidal inputs with a fixed amplitude and phase as 0 for both the full and Branch reduced models. PC firing changes dynamically with different frequency changes. Averaged PC spiking responses over many cycles of inputs show that PC firing is well modulated and fitted with a wide range of sinusoidal input frequencies for both the full and Branch reduced models (Figure 9B) for the amplitude of 25 Hz.

PC output amplitudes can be characterized by fitted sinusoidal functions, which shows that the PC output amplitude of this modulation is equal to the PC input amplitude up to 3 Hz for three typical PCs in the full models (Figure 9C). Then there is a small fluctuation of amplitude changes. Overall, these results are captured by reduced models under different schemes. The only exception is for the guinea-pig, shown in the Elect method (Figure 9C). In addition, the modulation phase changes are in phase with PF inputs at most of the frequencies (Figure 9D) for both the full and reduced models across the guinea-pig, mouse, and rat.

However, when the PF input amplitude is 50 Hz, the PC output amplitude can only be modulated equally at sinusoidal low frequencies below 3Hz and decays for larger frequencies, but the amplitude of the guinea-pig decays faster than the mouse and rat (Supplementary Figure 12). Moreover, for the Elect reduced model, the PC output amplitude is much less than the input amplitude at all frequencies (Supplementary Figure 12).

Therefore, the PC firing could be modulated by simplifying the morphological structure at low amplitude when the sinusoidal frequency changes. However, the firing rate of the guinea-pig PC could not be modulated at high amplitude in the Elect reduced model when the frequency changes. We conclude that the Elect model, which has the highest degree of simplification, makes it difficult to be modulated at high amplitudes. Together with the previous results, we believe that the complex morphological structure contributes to PC firing modulation with given sinusoidal PF inputs.

### 3.5. Inhibition Effect on PC Coding

Purkinje cells are the sole output of the cerebellar cortex. So far, we only consider the effects of excitatory inputs on PCs coding ability, since they receive the only excitatory signals of parallel fibers from granule cells which are the only output of granular layer. However, PCs also receive direct inhibitory input from several classes of molecular layer interneurons (Barmack and Yakhnitsa, 2008; He et al., 2015; Brown et al., 2019), which contribute the firing activities of PCs significantly as well (Brown et al., 2019).

**Figure 10:**
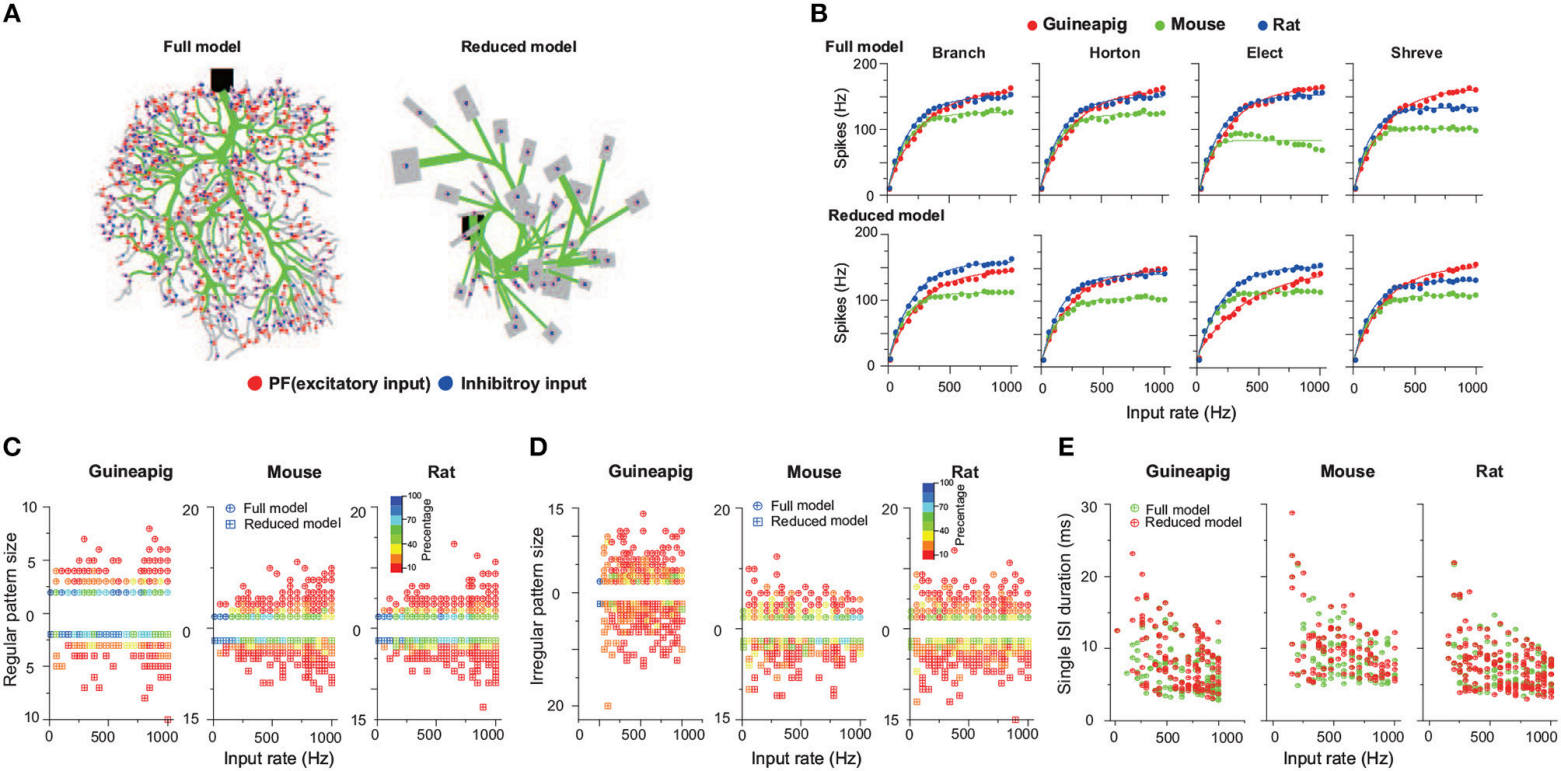
PC firing activities with 500 inhibitory and 1,000 excitatory inputs in Poisson stimulation. **(A)** Full (left) and Branch (right) models receive excitatory (red) and inhibitory (blue) synapses. **(B)** Comparison of firing response curves of three example PCs from the guinea-pig, mouse and ratt under four reduction schemes, Branch, Horton, Elect and Shreve, respectively. **(C)** Statistics of regular pattern size across a range of Poisson stimulation for the guinea-pig, mouse, and rat in the full and Branch models. Percentage of different size is indicated by different colors. **(D)** Similar as **(C)**, but for irregular patterns. **(E)** Single ISI duration across a range of Poisson stimulation.

Therefore, the effect of direct inhibition on PC firing activities were also studied by adding a population of inhibitory synaptic connections (See Methods). Indeed, we found there is a significant change of PC firing activities due to the inhibition input as shown in Figure 10 with the same three example PCs of the guinea-pig, mouse and rat under four reduction schemes under the Poisson stimulation. Compared to firing activities in Figure 4 and spike timing patterns in Figure 7 where there is only PF input, there is a systematic influence from the direct inhibitory input (See Supplementary Figures 6, 10, 11). Although the results with inhibition are comparable to those without inhibition for most of the cells, inhibitory input do play a functional role in adjusting firing activities, similar to experimental observations (Brown et al., 2019). The detailed performance with inhibitory inputs was computed in a similar way shown in Supplementary Table 1 for accuracy, Supplementary Table 2 for spike amplitude, and Supplementary Table 3 for spike width. Again, depending on the specific cells and species, there is a large diversity among different reduced methods.

## 4. Summary and Discussion

In this study, we investigated the coding capacity of Purkinje cells to excitatory parallel fiber input with different morphological reduction schemes. We proposed four reduction schemes to reduce the PC complex dendritic tree to a few components and tested these reduced models with 10 specific detailed PCs from three species of a guinea-pig, mouse and rat, respectively.

We showed, by performance evaluation and simulation, that reduced methods can balance accuracy and computational efficiency in different ways. We found that the Branch method has a better accuracy in most PCs, which is likely related to the preserved volume in the reduced method. We also found that there is no direct relationship between accuracy and simplification. In the Branch reduced model, PCs have the lowest level of simplification but only the tguinea-pig2 cell has the highest accuracy. In the Elect reduced model, the mouse1 cells show the highest accuracy and simplification at the same time. The Rat1 cell has the highest degree of simplification but presents the lowest accuracy. In addition, the computing efficiency is proportional to the degree of the simplification. Therefore, the diversity of different reduction methods for performance implies that one has to choose a proper method depending on the questions that needs to be addressed.

Compared to the Marosco model (Marasco et al., 2013), we found that the Horton and Shreve model has a better accuracy in most PCs, in particular, the Branch model is more accurate than the Marosco model across all PCs. Furthermore, the Elect model has a larger degree of simplification than the Marosco model in most PCs. We also found that the accuracy of the spike shape for four reduced models is higher than the Marosco model in most PCs. However, no single method can achieve both good accuracy and simplification at the same time. This suggests that there is a trade-off of morphology reduction between accurate firing activity and efficient runtime of stimulation, which may require more systematic investigations at the level of the neuronal network.

Precise firing coding is a key property of Purkinje cells in the cerebellum that is mainly used to control the high-precise motor patterns with millisecond timescale (Amir and Zee, 2011; Ostojic et al., 2015). We found that the mouse and rat PCs can respond to generate higher firing rates than guinea-pig PCs with the same stimulation. This may imply that rats and mice are able to react on a millisecond timescale better than guinea-pigs and may have better performance for precise temporal control of motor-related tasks and conditioned behaviors. It is worth noting that the animal species are not uniquely represented by encoding of firing rates since the same species have different rate coding due to their differences in morphology. However, the detailed mechanisms of why different species show different dynamic behaviors during reduction remain unclear. More evidence is needed to confirm and extend the current conclusion in the future.

In addition to firing rate coding, timing coding can be represented in PCs spiking patterns. With respect to simple spikes of PCs in response to PF excitatory inputs, PCs generate more regular spike trains than the stimulation sequences of the Poisson process. For stimulation of the renewal process, PF input sequences are more regular than PC spike trains at low frequencies. In contrary, PC spike trains are more regular than stimulation sequences at high frequencies. Furthermore, rat and mouse PC spikes are more regular than guinea-pig at high frequencies. It is worth noting that increasing the rate of input results in a regularity in the mouse and rat but has no effect on the guinea-pig. Inter-spike intervals have a linear relationship with refractory periods following each spike (Guan et al., 2006). We found that the guinea pig has the most irregular refractory periods under the same stimulus condition (Figure 6). Thus, we suggest that regularity is presumably determined by the refractory period. Moreover, the mouse and rat have similar results of the proportion of regular patterns in reduced models, but the mouse is more regular. This phenomenon is likely to be determined by the refractory period.

We stress the importance of morphology because the morphology is known to define the feature of neuronal types and has significant influences on neuronal computation. A wide range of patterns of firing activity, dendritic processing and synaptic integration produced by differences in morphology determine the response of neurons to synaptic inputs (Mainen and Sejnowski, 1996; Cannon et al., 2010; Einevoll et al., 2013). Indeed, we found that differences in neuronal morphology determine the response of PCs to synaptic inputs with a variation of firing rate, firing timing, and firing patterns. Here we mainly consider the case of PF synaptic inputs, since adding inhibitory inputs does not change the current results. However, it should be noted that the dynamics of neurons in the cerebellum is driven by a large diversity of synapses (Zampini et al., 2016), and network dynamics can be reshaped by the various types of synaptic plasticities (Liu and Buonomano, 2009; Liu, 2011), future efforts are needed to study the effect of morphology on neuronal and network dynamics via different synaptic dynamics (Hering and Sheng, 2001; Yuste and Bonhoeffer, 2001);(Ostojic et al., 2015).

**Figure 11:**
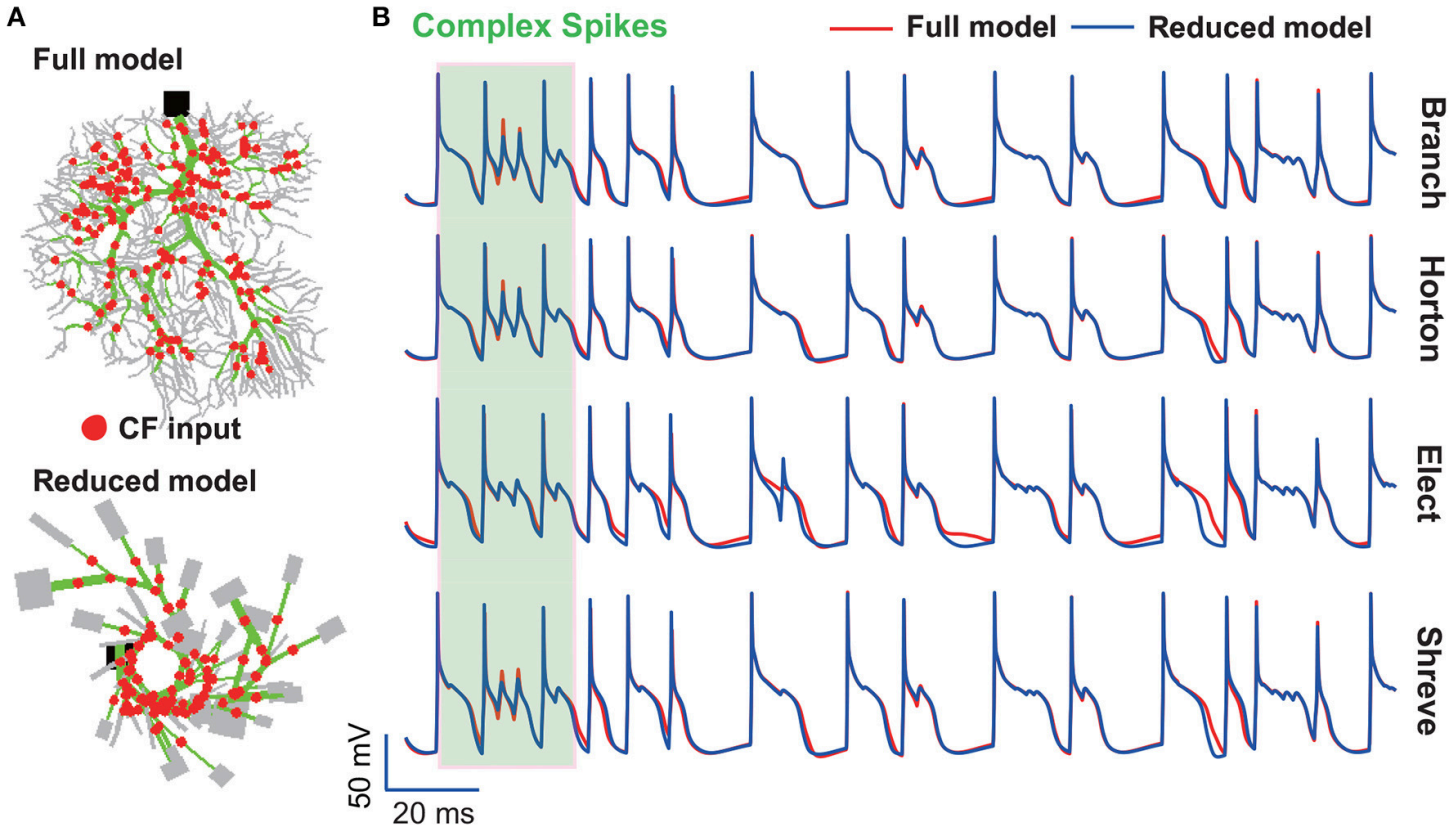
Complex spikes reproduced by reduced models. **(A)** Full and reduced morphology of the guinea-pig receiving CF (red dot) synapses. **(B)** Membrane potential traces recorded from the soma of guinea-pig PC in full (red) and four reduced models (blue). The shaded part indicates complex spikes. Poisson stimulation at 200 Hz.

It is well-known that Purkinje cells affect motor behavior via both simple and complex spikes (Khaliq and Raman, 2005; Harmon et al., 2017). We mainly addressed simple spikes in this study and future work is needed to systematically analyze complex spikes discharged by the PC. Simple spikes occur spontaneously and are modulated by synaptic inputs from granule cells. Complex spikes arise from climbing fibers and can induce plasticity of other afferents to Purkinje cells, therefore it plays the functional role of teaching or error signals during motor learning (Shogo and Medina, 2015; Titley et al., 2017). The granule cell inputs modulate simple spike firing up to 200 Hz, while climbing fibers trigger complex spikes at a remarkably low frequency (1 Hz) (Warnaar et al., 2015). In addition, complex spikes trigger prominent temporal pauses in the simple spike trains (Tang et al., 2017). While parallel fibers make synaptic contacts on spines in the spiny dendrite region, climbing fiber makes synaptic contact on the main and smooth dendrite regions (Schutter, 1999; Achard and Schutter, 2008). We mainly simplified the spiny dendrites of the reduced models and preserved most of the morphological structure of smooth and main dendrites. While parallel fibers make synaptic contact on spines in the spiny dendrite region, climbing fiber makes synaptic contact on the main and smooth dendrite regions. However, we found that membrane potential traces can be reproduced well in four reduced models at climbing fiber input (Figure 11). Thus, we speculate our reduced model can reproduce the properties of complex spikes very well. However, further systematic studies are needed to investigate the complex spikes.

Therefore, the reduced method can be redesigned to consider this feature of the regional difference. It is possible that spiny dendrites have no effect on evoked complex spikes, so they can be eliminated when simplifying the morphological structure.

The Purkinje cell is a very unique cell type in that it has a very large, perhaps the largest and most dense neuronal morphology. However, there are many other cell types defined in other areas of the brain, in which neuronal morphologies are very different as well. Therefore, it is possible that one has to design specific reduction schemes according to the uniqueness of neuronal morphology for each cell type. For instance, the typical morphology of layer V neurons in the cortex can generate specific dendritic spikes that are, in particular, important to be kept when their morphologies are reduced (Amsalem et al., 2018). Thus, one may not expect that there is a unique method for morphological reduction of all cell types in the neuronal system.

## Author Contributions

LA and JL conceptualization and project administration. LA, YT, RW, and HD formal analysis, Investigation, and methodology. LA, QP, QW, and JL funding acquisition. YT software and validation. LA, QW, and JL supervision. YT and JL visualization. LA, YT, and JL writing original draft.

### Conflict of Interest Statement

The authors declare that the research was conducted in the absence of any commercial or financial relationships that could be construed as a potential conflict of interest.
