# Supplementary material for: Coding Capacity of Purkinje Cells With Different Schemes of Morphological Reduction
Source: Front Comput Neurosci. 2019 May 15;13:29. doi: 10.3389/fncom.2019.00029 (PMC6530636; doi:10.3389/fncom.2019.00029)
Supplement: Supplementary file 1 [file Data_Sheet_1.pdf]

Coding Capacity of Purkinje Cells with Different Schemes of Morphological Reduction: Supplemental Figures

Lingling An†, Yuanhong Tang†, Quan Wang†, Qingqi Pei†, Ran Wei†, Huiyuan Duan†, Jian K. Liu‡

†School of Computer Science and Technology, Xidian University, Xi'an, China

‡Centre for Systems Neuroscience, Department of Neuroscience, Psychology and Behaviour, University of Leicester, Leicester, UK

Correspondence:: an.lingling@gmail.com, jian.liu@leicester.ac.uk

TABLE I

ACCURACY OF REDUCED MODELS WITH 500 INHIBITION INPUT. VALUES ARE MEAN ± STD. STD IS CALCULATED FROM 21 SETS OF POISSON STIMULATION FREQUENCY FROM 10 TO 1K HZ.

	**Guinea-pig1**	**Guinea-pig2**	**Guinea-pig3**	**Mouse1**	**Mouse2**	**Mouse3**	**Mouse4**	**rat1**	**rat2**	**rat3**
**Branch**	**0.957 ± 0.016**	0.927 ± 0.032	0.962 ± 0.020	0.926± 0.045	0.922± 0.042	**0.989± 0.008**	**0.980± 0.015**	0.970± 0.017	0.935± 0.021	0.953± 0.030
**Horton**	0.949 ± 0.018	**0.929 ± 0.023**	**0.971 ± 0.012**	0.880± 0.054	0.914± 0.039	0.971± 0.021	0.937± 0.027	0.967± 0.023	0.946± 0.016	**0.961± 0.021**
**Elect**	0.867 ± 0.034	0.836 ± 0.050	0.865 ± 0.041	0.836± 0.087	0.881± 0.058	0.958± 0.032	0.912± 0.026	**0.977± 0.024**	0.744± 0.065	0.915± 0.025
**Shreve**	0.946 ± 0.019	0.919 ± 0.030	0.966 ± 0.013	**0.955± 0.026**	**0.932± 0.034**	0.985± 0.014	0.980± 0.012	0.964± 0.018	**0.977± 0.017**	0.919± 0.041

TABLE II

THE CHANGE OF SPIKE AMPLITUDE (MV) IN REDUCED MODELS WITH 500 INHIBITION INPUTS. VALUES ARE MEAN ± STD. STD IS CALCULATED FROM 21 SETS OF POISSON STIMULATION FREQUENCY FROM 10 TO 1K HZ.

	**Guinea-pig1**	**Guinea-pig2**	**Guinea-pig3**	**Mouse1**	**Mouse2**	**Mouse3**	**Mouse4**	**rat1**	**rat2**	**rat3**
**Branch**	**1.4 ± 0.9**	2.7 ± 1.4	2.7 ± 1.3	0.4 ± 0.2	**3.4 ± 1.2**	**0.1 ± 0.2**	0.2 ± 0.1	2.2 ± 0.9	2.5 ± 0.9	0.6 ± 0.3
**Horton**	2.3 ± 0.9	2.2 ± 0.6	**1.5 ± 0.6**	0.5 ± 0.2	3.6 ± 1.4	0.4 ± 0.2	**0.1 ± 0.1**	0.8 ± 0.3	**0.3 ± 0.5**	**0.5 ± 0.2**
**Elect**	2.1 ± 2.1	**1.3 ± 0.7**	**1.4 ± 0.9**	0.5 ± 0.1	6.6 ± 2.1	0.9 ± 0.6	3.8 ± 0.1	**0.4 ± 0.6**	1.3 ± 0.7	2.1 ± 0.9
**Shreve**	3.1 ± 1.1	3.0 ± 1.1	2.1 ± 0.9	**0.1 ± 0.1**	3.4 ± 1.3	0.3 ± 0.2	0.6 ± 0.3	1.2 ± 0.3	0.3 ± 0.7	1.3 ± 0.5

TABLE III

THE CHANCE OF SPIKE WIDTH (MS) IN REDUCED MODELS WITH 500 INHIBITION INPUTS. VALUES ARE MEAN ± STD. STD IS CALCULATED FROM 21 SETS OF POISSON STIMULATION FREQUENCY FROM 10 TO 1K HZ.

	**Guinea-pig1**	**Guinea-pig2**	**Guinea-pig3**	**Mouse1**	**Mouse2**	**Mouse3**	**Mouse4**	**rat1**	**rat2**	**rat3**
**Branch**	0.23 ± 0.29	0.52 ± 0.23	0.27 ± 0.18	0.20 ± 0.07	1.45 ± 0.92	0.18 ± 0.12	0.3 ± 0.13	0.31 ± 0.49	0.56 ± 0.25	**0.09± 0.1**
**Horton**	0.29 ± 0.43	0.69 ± 0.36	**0.16±0.23**	0.26 ± 0.07	1.1 ± 0.93	0.1 ± 0.11	0.7 ± 0.3	**0.1 ± 0.1**	0.36 ± 0.21	0.1 ± 0.12
**Elect**	0.68 ± 0.52	1.07 ± 1.02	0.64 ± 0.68	**0.1 ± 0.12**	**0.6 ± 0.6**	0.36 ± 0.48	0.43 ± 0.49	0.24 ± 0.37	0.55 ± 0.32	0.71 ± 0.9
**Shreve**	**0.21±0.14**	**0.49±0.34**	0.19 ± 0.34	0.19 ± 0.05	0.97 ± 0.8	**0.1 ± 0.1**	**0.25± 0.2**	0.44 ± 0.47	**0.26± 0.18**	0.34 ± 0.39

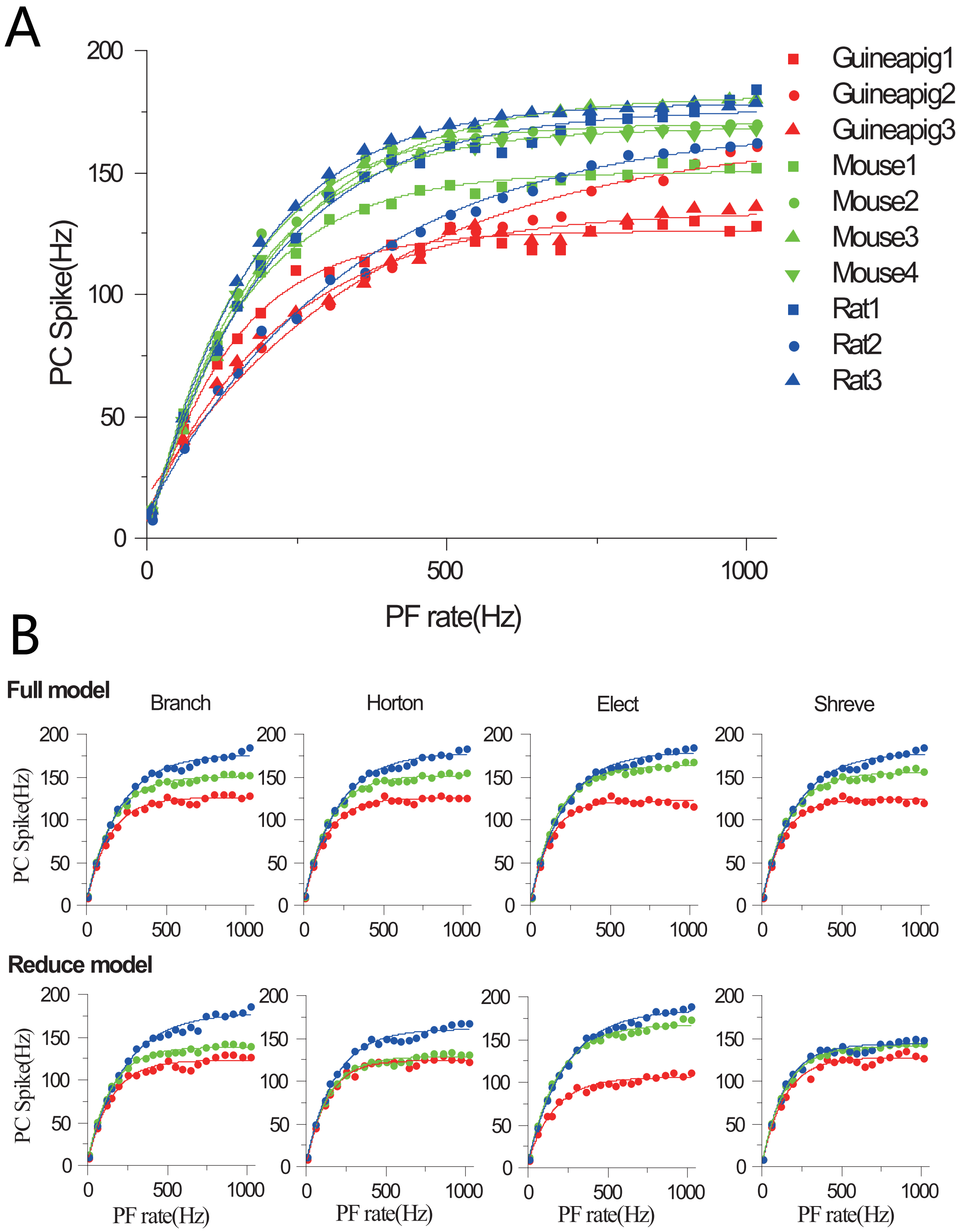

Fig. 1. PC firing response in full and reduced model. (A) Firing response curves of ten PCs with full morphology with renewal process stimulation frequencies 10Hz to 1000Hz. (B). Comparison of firing response curves of three example PCs from guinea-pig, mouse and rate under four reduction schemes, Branch, Horton, Elect and Shreve, respectively.

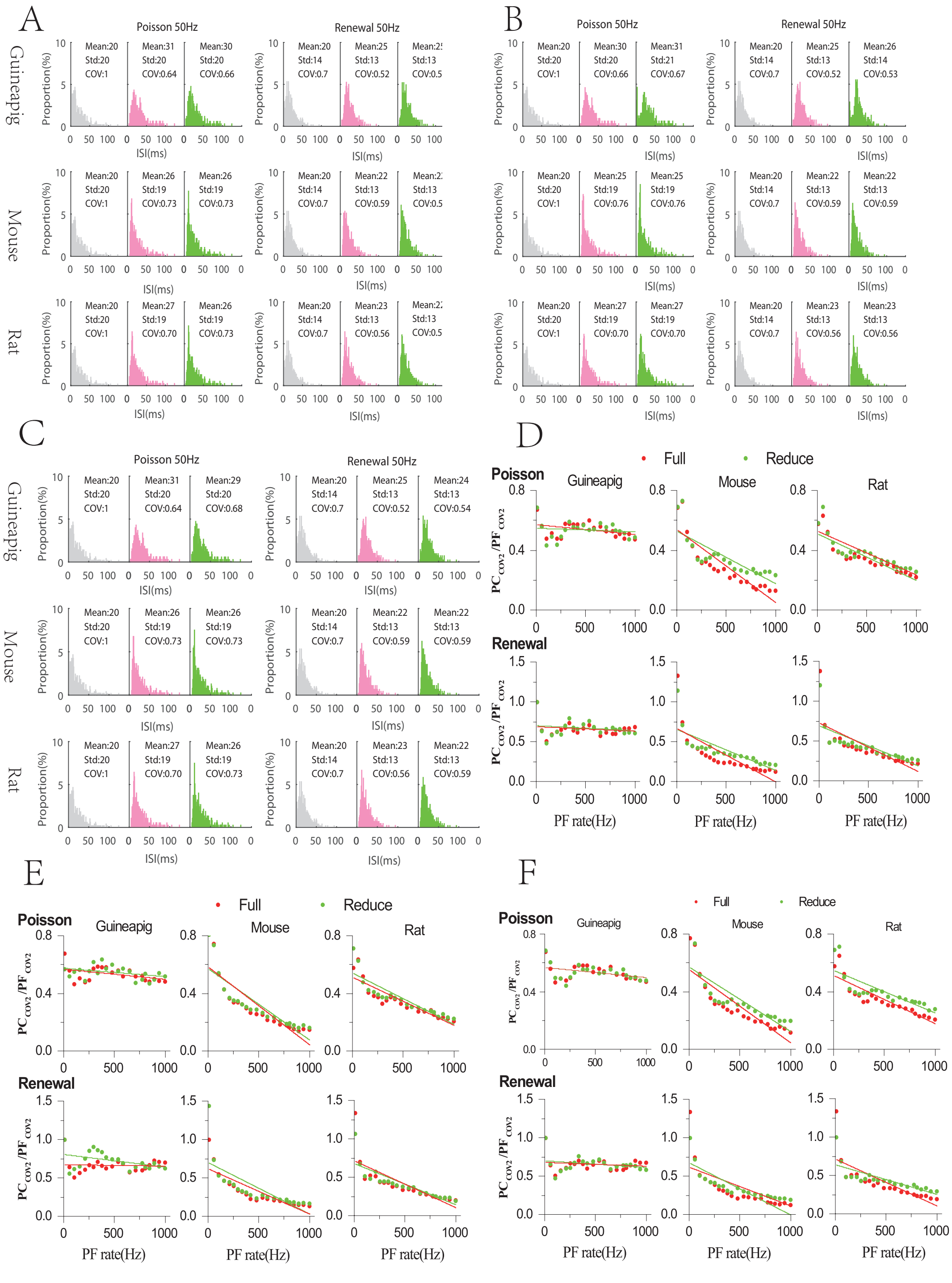

Fig. 2. PC temporal response under Poisson and renewal stimulation. (A), (B) and (C) are Horton, Elect and Shreve reduced model, respectively. ISI distribution of spike trains from PF (gray), PC full model (light red) and PC reduced model (green) under Poisson and renewal process stimulation for guinea-pig (top), mouse (middle), rat (bottom). All stimulation frequencies are 50Hz for 10 seconds. P> 0.1, Wilcoxon Rank-sum test. (D), (E) and (F) are Horton, Elect and Shreve reduced model, respectively. PC_{cov2}/PF_{cov2} showing the regularity between PF inputs and PC outputs for full (red) and reduced model (green) of guinea-pig, mouse and rat. Stimulations are Poisson and renewal process with different frequencies from 10 to 1K Hz.

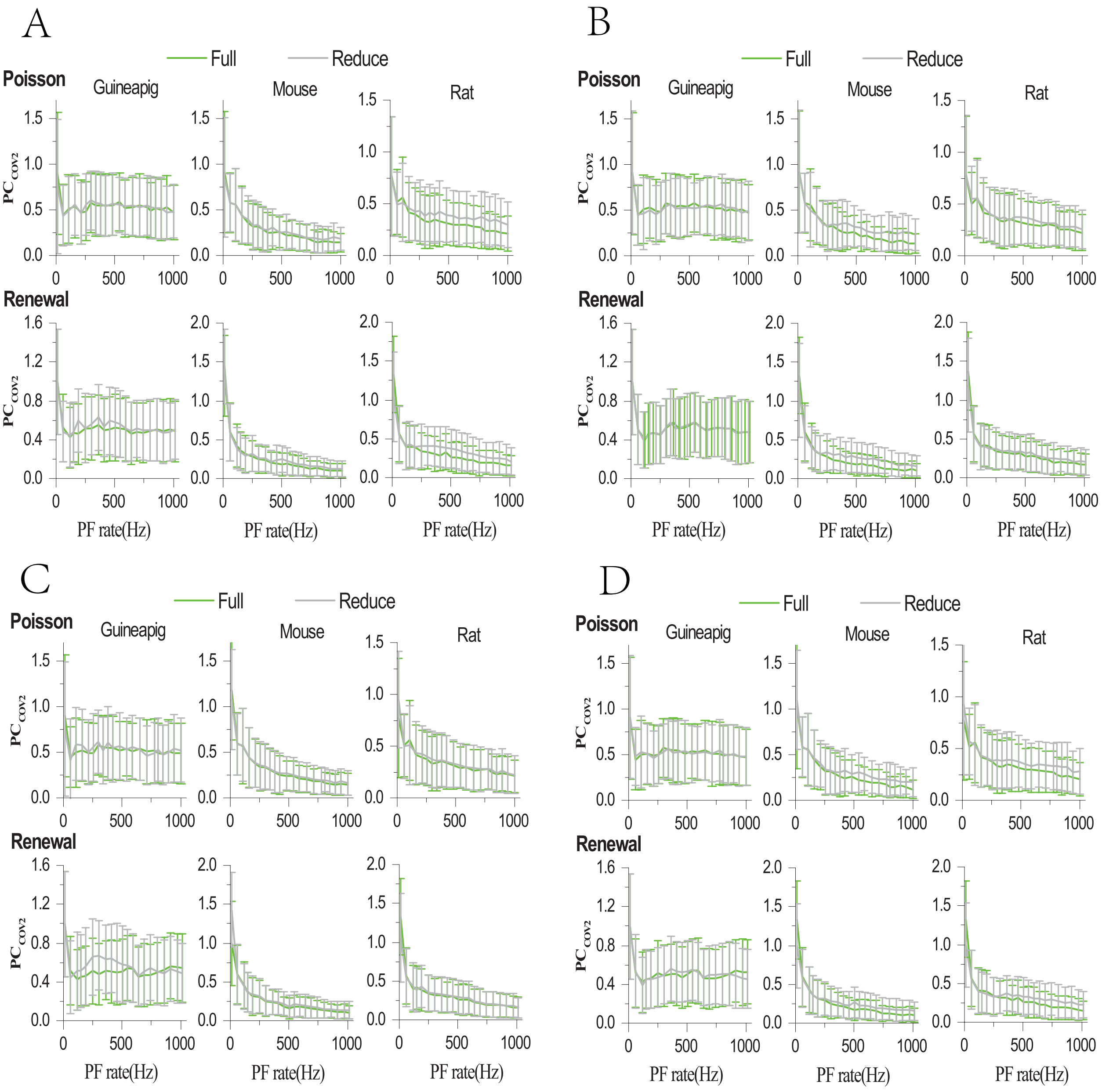

Fig. 3. (A)(Branch), (B)(Horton), (C)(Elect) and (D)(Shreve) show the $mean \pm std$ of cov_2 of the PC spike trains in full (green) and reduced (gray) model with Poisson and Renewal stimulation frequencies 10Hz to 1KHz.

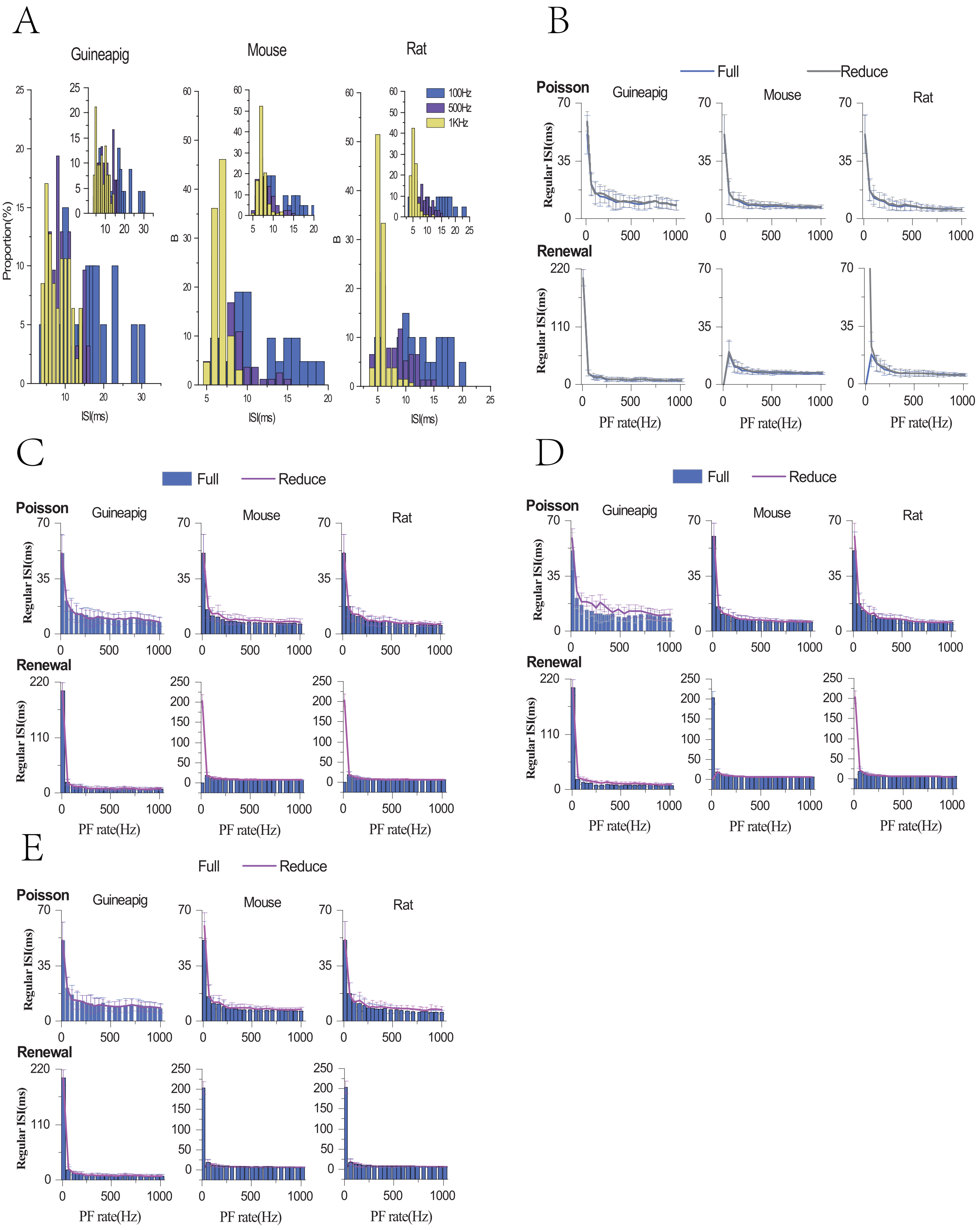

Fig. 4. PC spiking patterns. (A). The mouse PC spiking regular pattern for 100Hz, 500Hz and 1KHz with Poisson stimulation in full and Branch reduced model(inset). (B)(Branch), (C)(Horton), (D)(Elect) and (E)(Shreve) show $mean \pm std$ of regular patterns with Poisson and renewal stimulation in full and reduced modes.

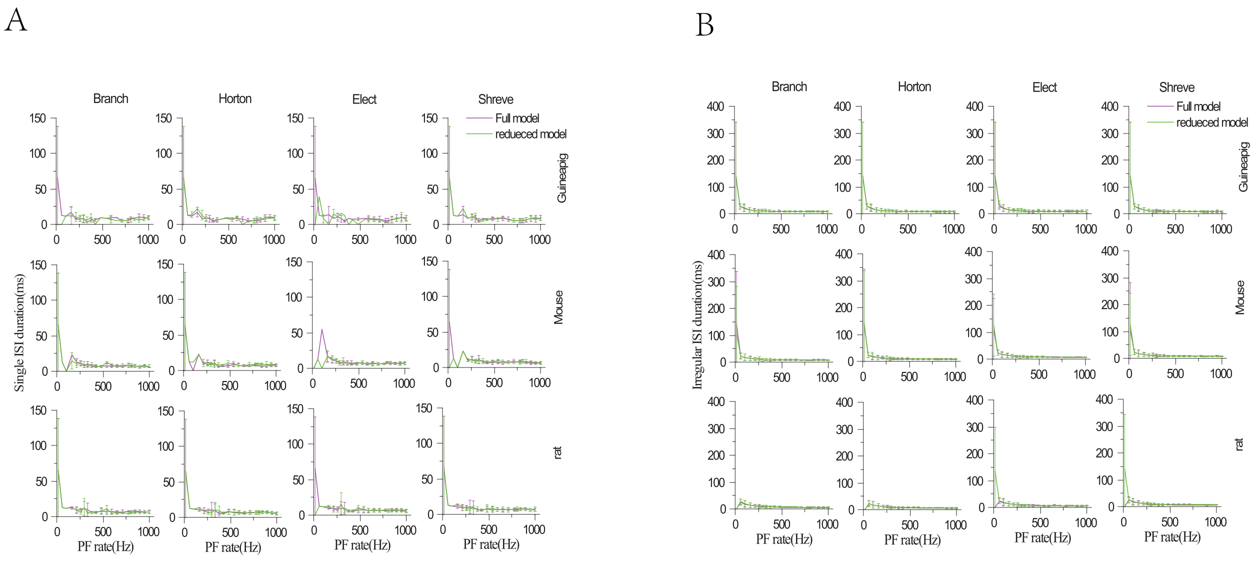

Fig. 5. PC spiking patterns. (A). Single ISI duration ($mean \pm std$) with Poisson stimulation for guinea pig (top), mouse (middle) and rat (bottom) in full and reduced models. (B) Irregular patterns.

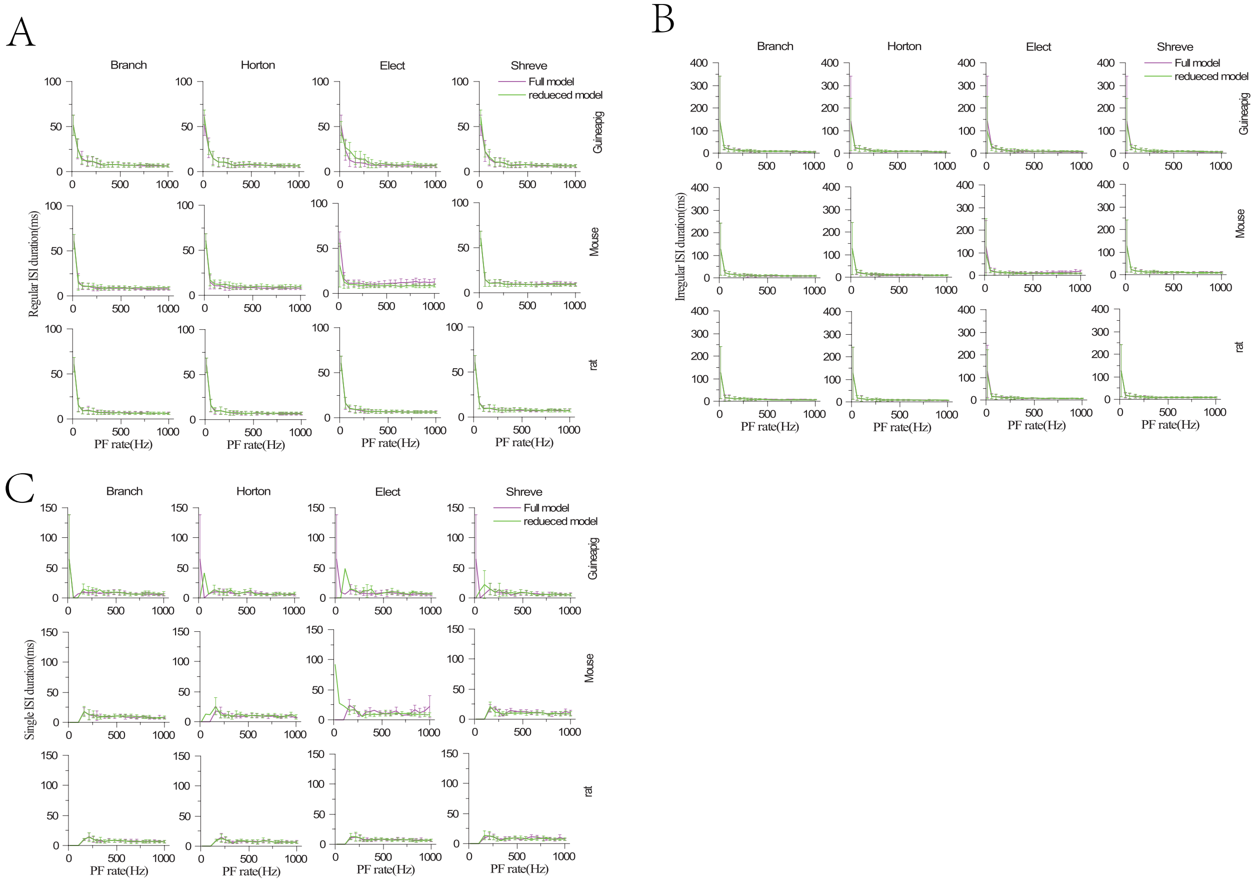

Fig. 6. PC spiking patterns with inhibition input. (A) Regular pattern ISI duration ($mean \pm std$) with Poisson stimulation for guinea pig (top), mouse (middle) and rat (bottom) in full and reduced model. (B) Irregular pattern ISI duration distribution. (C) Single ISI duration distribution.

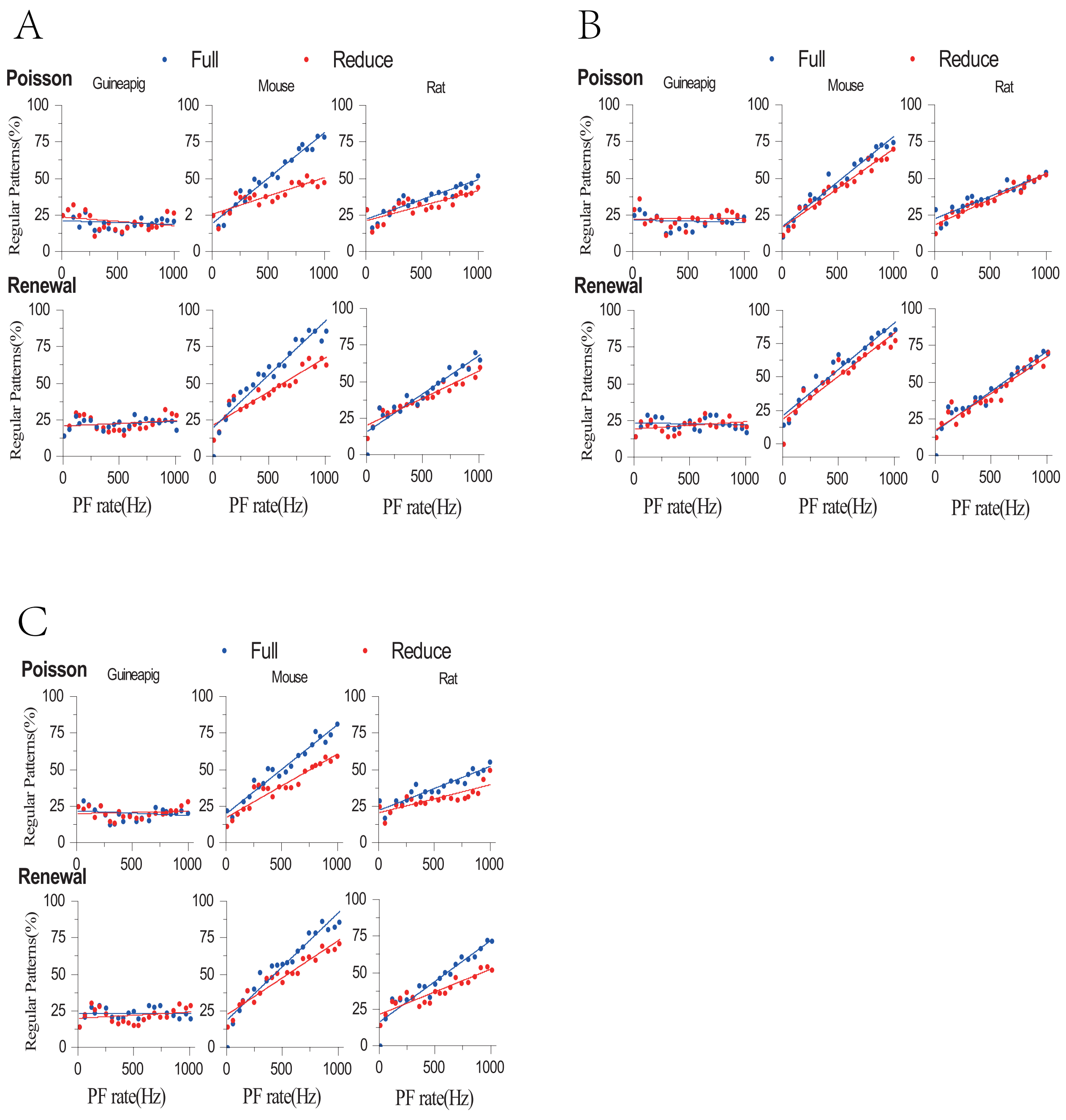

Fig. 7. Percentage of regular patterns in full (blue) and reduced model (red) with Poisson and renewal stimulation at different stimulation frequencies 10Hz to 1KHz. (A), (B) and (C) show Horton, Elect and Shreve reduced model respectively.

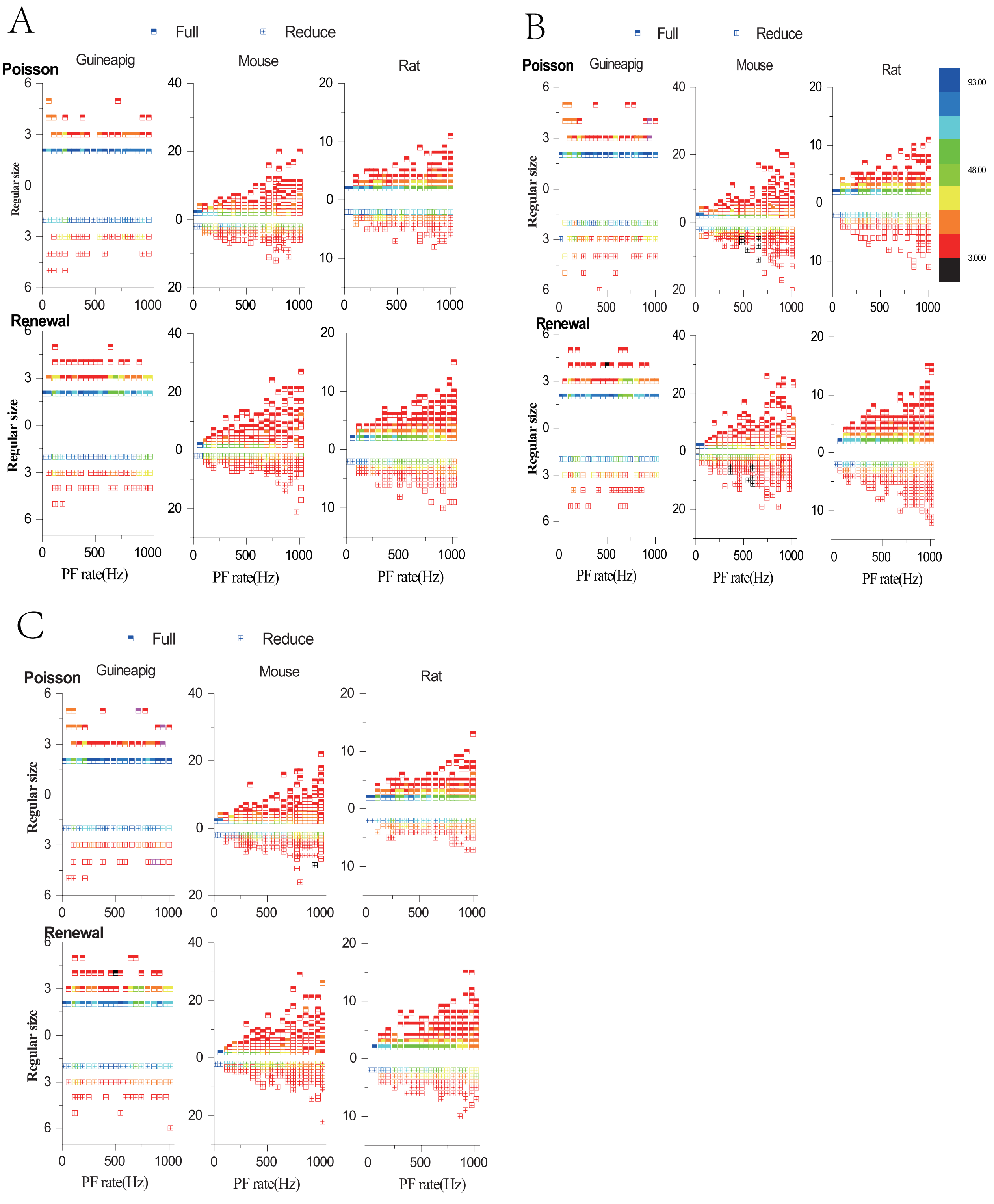

Fig. 8. Statistics of regular pattern size across a range of Poisson and renewal process stimulation for guinea-pig, mouse, and rat in full and reduced model. Percentage of different size is indicated by different colors, as there are more patterns in higher frequency. (A), (B) and (C) show Horton, Elect and Shreve reduced model respectively.

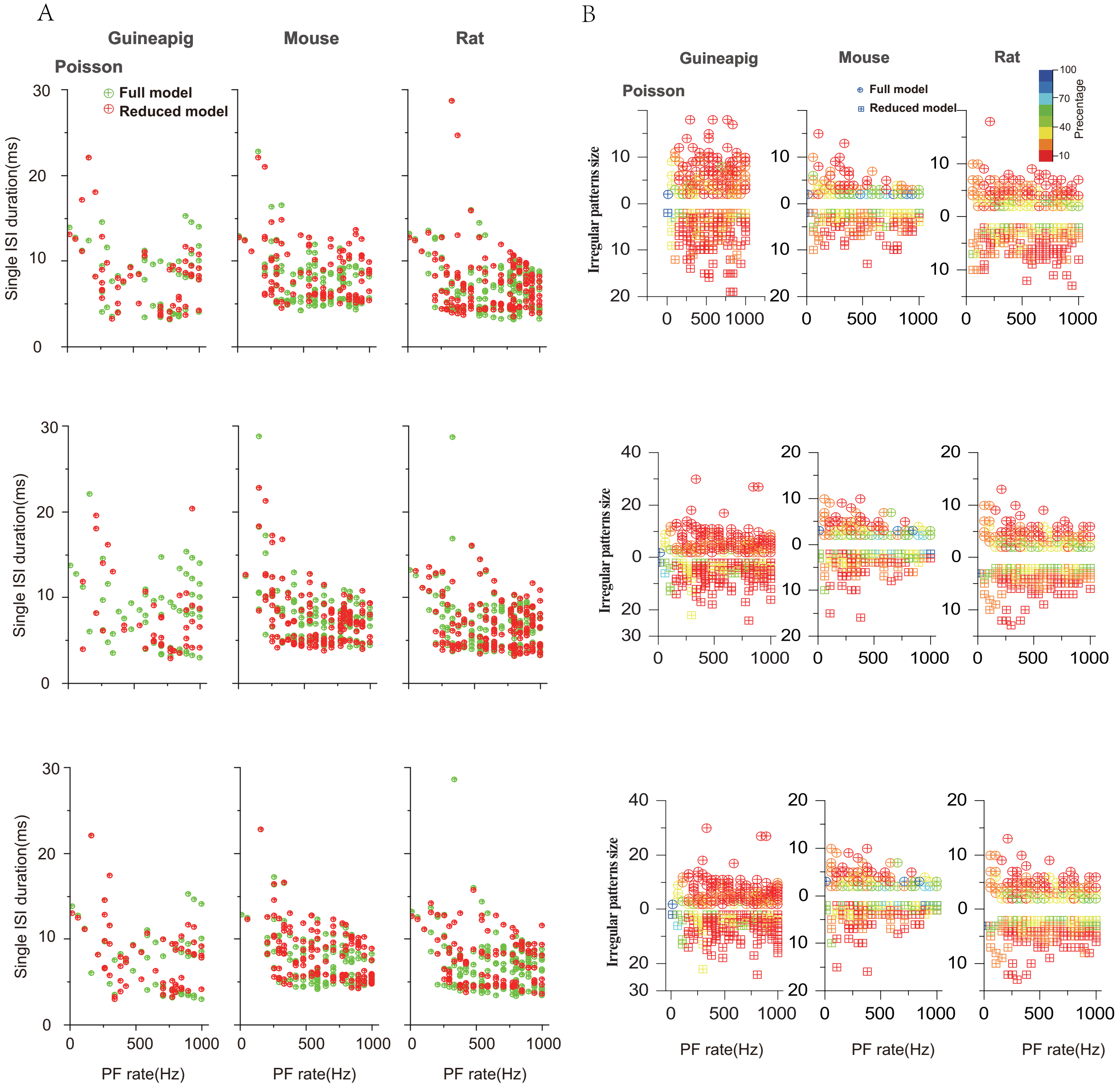

Fig. 9. Statistics of single ISI duration distribution and irregular pattern size across a range of Poisson stimulation for guinea-pig, mouse, and rat in both full and reduced model. Percentage of different size is indicated by different colors. Single size (A), irregular size (B) of Horton (top), Elect (middle) and Shreve (bottom) reduced model, respectively.

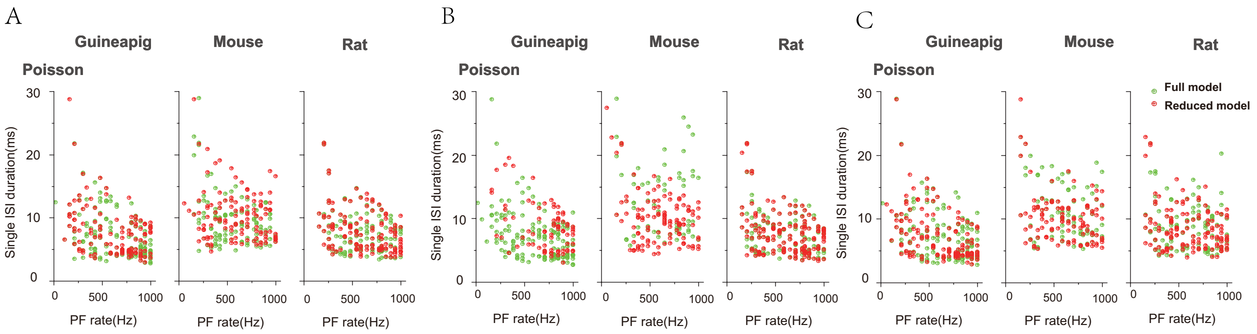

Fig. 10. Statistics of single ISI duration distribution across a range of Poisson stimulation for guinea-pig, mouse, and rat in full and reduced model with inhibition input. (A) Single ISI duration distribution of Horton reduced model. (B) Single ISI duration distribution of Elect reduced model. (C) Single ISI duration distribution of Shreve reduced model.

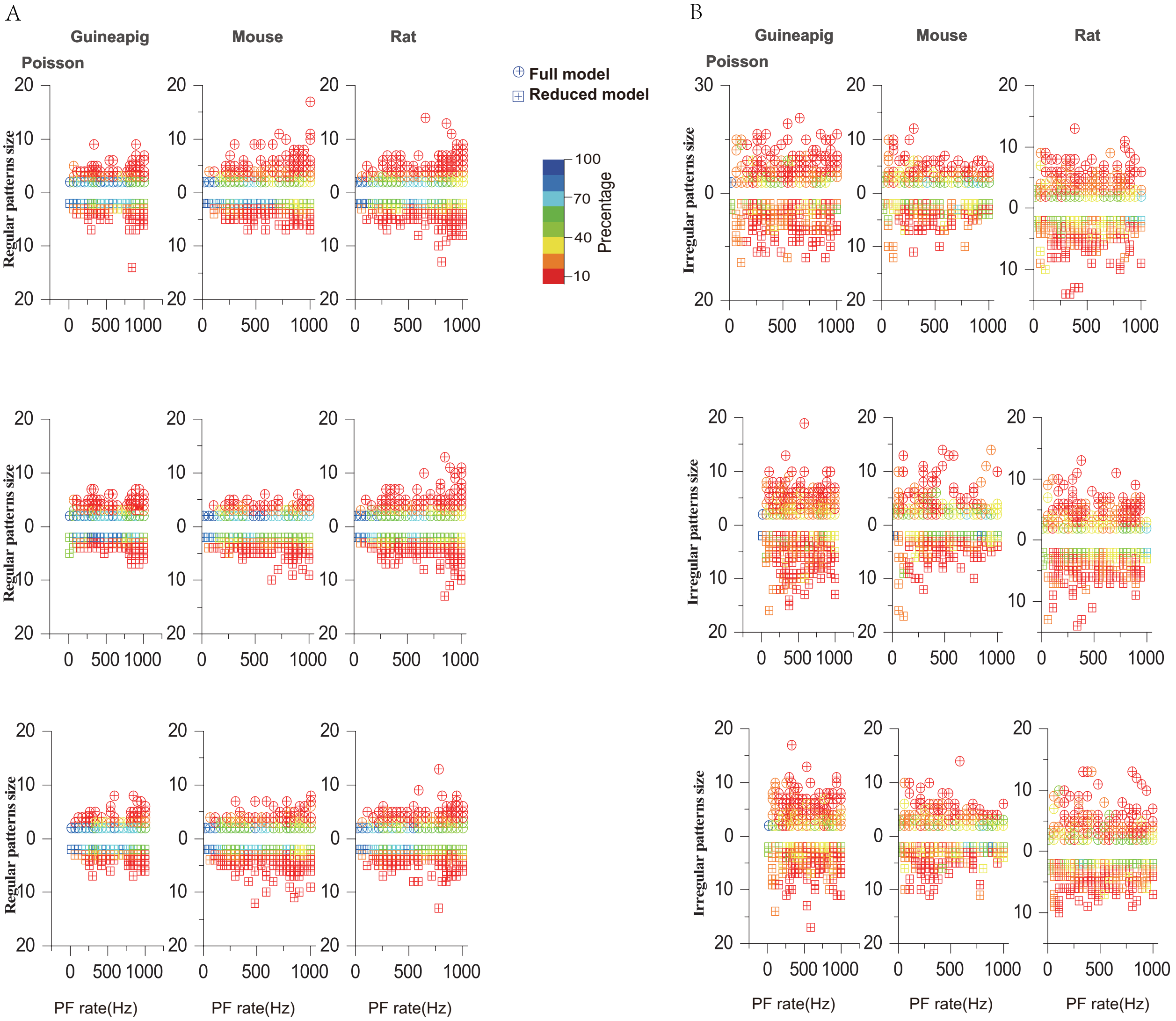

Fig. 11. Statistics of regular pattern size and irregular pattern across a range of Poisson stimulation for guinea-pig, mouse, and rat in full and reduced model with inhibition input. Percentage of different size is indicated by different colors. (A) Regular pattern size of Horton (top), Elect (middle) and Shreve (bottom) reduced model. (B) Irregular pattern size of Horton (top), Elect (middle) and Shreve (bottom) reduced model.

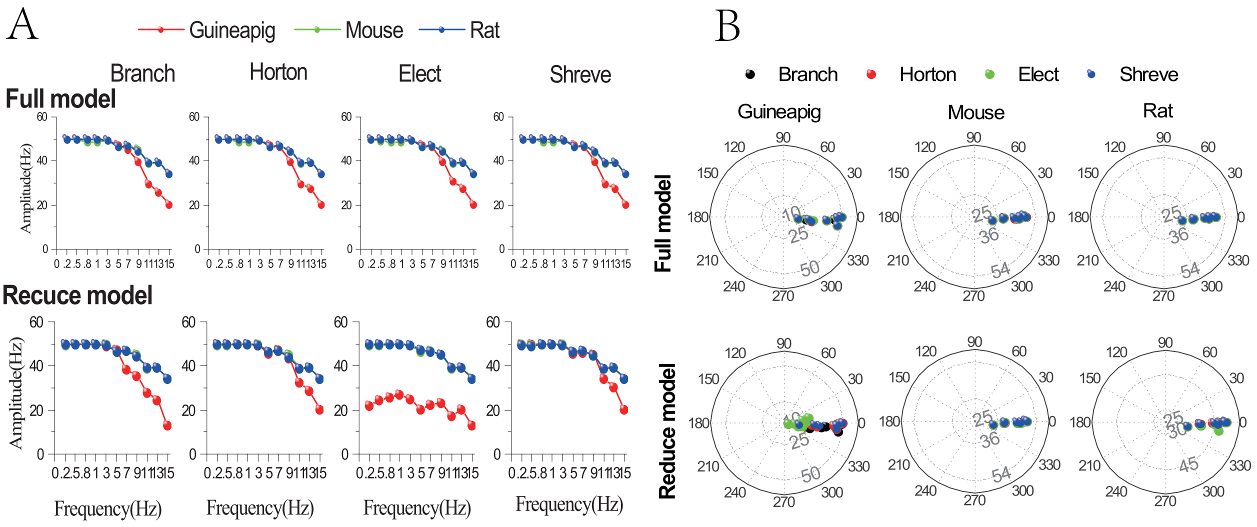

Fig. 12. (A) Modulation amplitudes of PC output in full and reduced model of four reduced schemes for guinea-pig (red), mouse (green), and rat (blue), respectively, at different PF input frequencies. (B) Similar to (A), but for phase change of PC firing modulation for full and reduced model of four reduce schemes. PF sinusoidal stimulation amplitude is 50Hz.
